# Synthesis of Imidazolidin-2-ones from *trans*-(*R*,*R*)-Diaminocyclohexane: A Statistical Analysis-Based Pseudo-Multicomponent Protocol

**DOI:** 10.3390/molecules30071415

**Published:** 2025-03-22

**Authors:** Catalina Hoyos-Orozco, Lili Dahiana Becerra, Diego Quiroga

**Affiliations:** Bioorganic Chemistry Laboratory, Facultad de Ciencias Básicas y Aplicadas, Universidad Militar Nueva Granada, Km 2, Cajicá 250247, Colombia; scatalinahoyoso@gmail.com (C.H.-O.); lili.becerra@unimilitar.edu.co (L.D.B.)

**Keywords:** imidazolidin-2-one, diaminocyclohexane, Schiff base reduction, CDI, statistical analysis

## Abstract

A pseudo-multicomponent one-pot protocol for the synthesis of 1,3-disubstituted imidazolidin-2-one is described, employing *trans*-(*R*,*R*)-diaminocyclohexane for the in situ formation of the Schiff base, followed by reduction to produce the respective diamine and cyclization with carbonyldiimidazole (CDI). This approach utilizes statistical analysis to optimize the reaction conditions, allowing a pseudo-multicomponent protocol to be proposed. The developed method demonstrates sustainability, efficiency, and potential applications in green chemistry, achieving yields ranging from 55% to 81%. This represents a significant advance in synthesizing heterocyclic compounds with biological and pharmacological applications.

## 1. Introduction

**Scheme 1:**
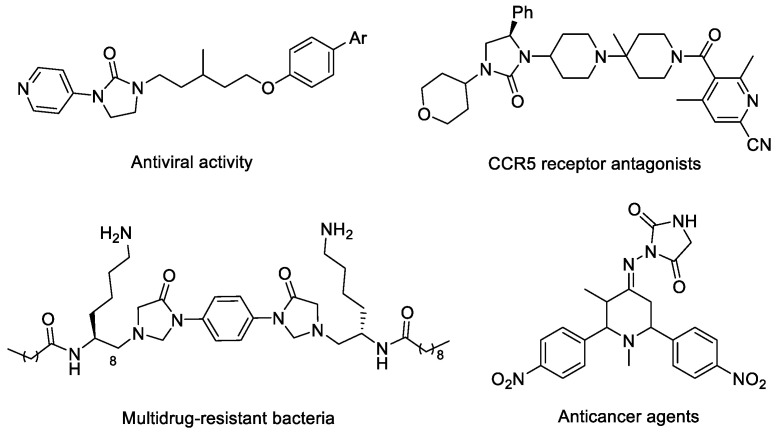
Imidazolidines and their derivatives, along with reported biological activities.

Imidazolidines and their derivatives are versatile heterocyclic compounds with significant biological and pharmacological applications, serving as key structural components of several FDA-approved drugs, including emicerfont, imidapril, azlocillin, ethotoin, nitrofurantoin, and dantrolene. Given their broad therapeutic potential, research continues to explore new compounds based on this scaffold. Swain and Mohanty [1] reviewed imidazoline-2-one and imidazoline-2,4-dione derivatives with potent antiviral activity against HIV, dengue, hepatitis C, and enteroviruses. Building on this, Rotstein et al. [2] developed CCR5 receptor antagonists using imidazolidinone derivatives, with the *R*-enantiomer exhibiting the best antiviral activity and pharmacokinetics in rat studies. In addition to their antiviral properties, these compounds have also been explored for their antimicrobial potential. Wang et al. [3] synthesized *bis*-cyclic imidazolidin-4-ones effective against multidrug-resistant bacteria, while Marinov et al. [4] reported imidazoline-2,4-dione derivatives with activity against Gram-positive and Gram-negative bacteria, yeasts, and molds. Their biological relevance extends further into cancer research, where Dewangan et al. reviewed the role of imidazolidinone derivatives as anticancer agents, exploring their synthesis and structure–activity relationship [5]. AbdulJabar et al. [6] demonstrated anti-liver and breast cancer activity, while Nafie et al. [7] reported cytotoxic effects against HepG2 cells in 2-thioxo-4-imidazolidinone-derived compounds. Imidazolidine derivatives have also been reported to exhibit antioxidant activities [8,9] (Scheme 1).

**Scheme 2:**
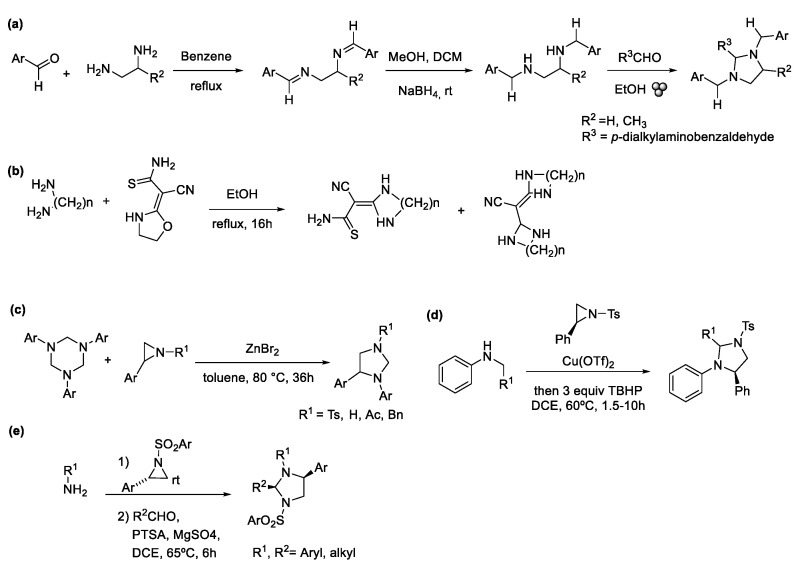
Reported synthetic methodologies to access to imidazolidine systems. (**a**) synthesis of 1,2,3-trisubstituted imidazolidines, (**b**) synthesis of imidazolines and tetrahydropyrimidines, (**c**) synthesis of 1,2,3,4-tetrasubstituted imidazolidines, (**d**) synthesis of 1,3-imidazolidines, (**e**) synthesis of 2,3-disubstituted-4-aryl-1-(arylsulfonyl)imidazolidines.

The synthesis of five-membered nitrogenous heterocycles, particularly those featuring nitrogen atoms at the 1- and 3-positions, typically involves methods such as cyclocondensation, radical cyclizations, metal-catalyzed coupling, and cycloadditions [10]. Many of these traditional approaches rely on 1,2-diamines as starting materials (Scheme 2). For example, Husain et al. [11] employed Schiff base formation between 1,2-diamines and aromatic aldehydes, followed by reduction and cyclocondensation under reflux to synthesize 1,2,3-trisubstituted imidazolidines (Scheme 2a). Similarly, Beyzaei et al. [12] synthesized imidazolines and tetrahydropyrimidines using diaminoalkanes under reflux or solvent-free conditions (Scheme 2b). Tu et al. [13] utilized a 1,3-dipolar cycloaddition between 1,3,5-triazinanes and aziridines, catalyzed by zinc bromide, achieving high yields of up to 92% (Scheme 2c). Sengoden et al. [14] employed a domino reaction with copper(II) and TBHP to generate 1,3-imidazolidines via aziridine ring opening, C–H activation, and C–N bond formation, yielding high enantiomeric excess (Scheme 2d). Tarannum et al. [15] developed a one-pot stereospecific synthesis route for imidazolidines, using aziridine ring opening, amine reaction, and intramolecular cyclization with aldehydes, reaching up to 92% yield with excellent stereoselectivity (Scheme 2e).

While these traditional synthetic routes are effective, they often involve multiple steps, specific reagents, and harsh reaction conditions. These challenges have spurred interest in more efficient and sustainable synthetic methodologies. In this regard, multicomponent reactions (MCRs) have emerged as a highly attractive alternative, offering a more sustainable and efficient approach by minimizing the need for isolated intermediates and reducing the environmental impact [16,17]. MCRs are domino-type one-pot processes that involve the sequential combination of three or more reactants in the same reactor to produce a target compound [18]. This strategy offers significant advantages in terms of time and resource efficiency, particularly for synthesizing complex heterocyclic compounds with multiple functional groups. Notably, MCRs enable the direct assembly of compounds with minimal waste, a key consideration in green chemistry. Furthermore, pseudo-MCRs [19], which also involve a combination of three or more reactants, further enhance synthetic diversity by incorporating components that participate in multiple steps within the reaction mechanism, thereby allowing for the efficient generation of libraries of complex compounds in fewer steps. While pseudo-MCRs may appear to involve fewer distinct reagents, their ability to employ repeated reactants in varied reaction steps opens avenues for more nuanced control over molecular complexity. Given these compounds’ significant biological and pharmaceutical relevance, the application of MCRs and pseudo-MCRs to synthesizing imidazolidines and related heterocycles is particularly noteworthy. Recent advancements have demonstrated the practicability of MCRs in improving yields and functional group compatibility, even under milder conditions [20,21,22,23,24]. These developments highlight the significant progress made in utilizing MCRs for the efficient and sustainable synthesis of bioactive compounds, underscoring their potential application in diverse fields, including pharmaceuticals, agrochemicals, and material science.

**Figure 1:**
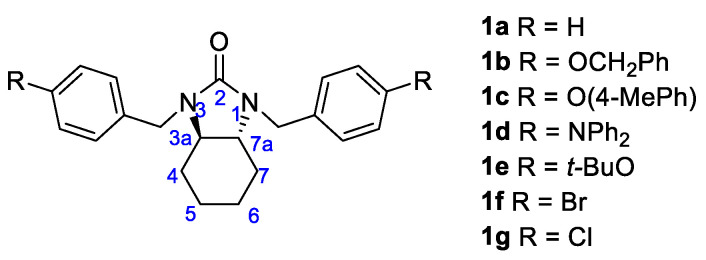
Chemical structure of the synthesized compounds **1a**–**g** (numeration of carbon atoms in perhydrobenzimidazole-2-one ring appears in blue).

In our research, we have focused on simplifying the synthesis of 1,3-disubstituted imidazolidin-2-ones from *trans*-(*R,R*)-1,2-diamino cyclohexane (Figure 1), utilizing a pseudo-multicomponent reaction strategy. This approach begins with the in situ formation of a Schiff base, followed by reduction and cyclization with carbonyldiimidazole (CDI), a reagent widely employed in the coupling of carboxylic acids and amines to form amides [11]. CDI offers several advantages, including its low cost, availability, and benign byproducts, such as carbon dioxide and imidazole, which are unlikely to cause issues in scaled-up reactions [25,26]. However, the slow reaction rates between aromatic amines and CDI intermediates have hindered the broader application of CDI in high-throughput environments, particularly within pharmaceutical manufacturing [11]. Recently, Prusinowska et al. reported significant advances in the control of the conformational dynamics of chiral macrocycles and polyamines with figuratively periodic structures by introducing urea functionalities, which was made possible through CDI [27].

Considering these previous findings, this study aims to leverage the advantages of pseudo-MCRs, alongside CDI’s unique reactivity, to create a more efficient, streamlined process for imidazolidine-2-one synthesis, providing a greener alternative to traditional methods. Thus, a statistical analysis of a series of experiments exploring the synthesis of 1,3-disubstituted imidazolidin-2-ones via a pseudo-multicomponent reaction, in which the Schiff base is formed in situ from *trans*-(*R,R*)-diaminocyclohexane, followed by reduction and cyclization with CDI, is presented. The results of these experiments are discussed below, illustrating the potential for MCRs and pseudo-MCRs to offer a transformative approach to the synthesis of nitrogenous heterocycles, further advancing the field of green and sustainable chemistry.

## 2. Results and Discussion

### 2.1. Optimization of the Two-Step Synthesis of Imidazolin-2-ones ***1a**–**g***

To synthesize imidazolidin-2-ones **1a**–**g**, initially, a multistep methodology was employed, which involved three stages: Schiff base formation, reduction to diamine, and cyclization via reaction with carbonyldiimidazole (CDI). The process began with the enantiomerically pure *trans*-(*R*,*R*)-diaminocyclohexane, obtained by basifying *trans*-(*R*,*R*)-diammoniumcyclohexane mono-*L*(+)-tartrate [28] with a saturated sodium bicarbonate solution and extracting with dichloromethane. The first reaction, which involved the formation of the Schiff base **2a**, was carried out using methanol and THF as solvents, which are classified as medium- to high-polarity solvents. The reaction was carried out at reflux conditions (40 and 80 °C, respectively) and was continuously monitored by thin layer chromatography (TLC) using a mobile phase composed of a mixture of hexane and ethyl acetate in a proportion of 8:2 and silica gel as the stationary phase. The complete consumption of benzaldehyde, which was detected by TLC, made it possible to establish the reaction times in the first step. Subsequently, sodium borohydride (NaBH_4_) was added to reduce the Schiff base **2a**, followed by TLC until the total consumption of **2a** was confirmed. The results of each step are presented below (Table 1).

Using methanol as a solvent was suitable for forming the Schiff base **2a** and its subsequent reduction. The formation of the Schiff base **2a** did not require high temperatures, and the reaction time was relatively short. Furthermore, the reduction was also performed at 40 °C and for 30 min and showed a good yield. Compared to methanol, THF requires higher temperatures (80 °C) for both the Schiff base formation and the reduction. The reaction time for the reduction is also significantly longer (4 h versus 30 min for methanol). Despite these more extreme parameters, the yield obtained (74%) is almost equal to that of methanol (74%). However, the longer reaction time in THF could indicate slower reaction in this solvent.

Structural elucidation of compound **3a** was carried out by ^1^H and ^13^C nuclear magnetic resonance (NMR) spectroscopy. In the ^1^H NMR spectrum, several characteristic signals are observed: axial hydrogens of the cyclohexane system (Figure 1), marked as H4_axial_, H5_axial_, H6_axial_, H7_axial_, appear as a multiplet in the range of 1.14–1.02 ppm; equatorial hydrogens (H5_equatorial_, H6_equatorial_) occur at 1.75–1.70 ppm; and hydrogens attached to chiral centers (H3a, H7a) at 2.32–2.27 ppm. The signals corresponding to the diastereotopic benzylic hydrogens are observed as doublets at 3.91 and 3.66 ppm, with a coupling constant of 15.2 Hz. Furthermore, the aromatic hydrogens of the benzene ring occur as a multiplet in the range of 7.37–7.22 ppm. Regarding the ^13^C NMR spectrum, the chemical shifts reveal the presence of carbon atoms in specific positions: the methylene carbons of cyclohexane (C4 and C5) are found at 24.9 ppm, the methylene carbons (C4 and C7) at 31.1 ppm, and the chiral carbons (C3a and C7a) at 60.5 ppm. The benzylic methylene carbon is located at 50.5 ppm, and the aromatic carbons in the phenyl system exhibit chemical shifts at 140.2 ppm, 128.4 ppm, 128.1 ppm, and 126.9 ppm. These results are consistent with those reported in the literature [29].

The cyclization of diamine **1a** with CDI (Table 2) allowed evaluation of the influence of the solvent on the reaction yield. The reaction performed in toluene did not produce the desired compound, as the yield was less than 5% after 64 h. The low solubility of the reactants could explain this low yield in the apolar solvent, which limits the reactivity and hinders the formation of the main product. When tetrahydrofuran (THF) was used as the solvent at 20 °C, a higher yield was observed, reaching 21% after 64 h. However, this yield is still relatively low, suggesting that, although THF is more polar than toluene, it does not optimally favor the cyclization of the diamine with CDI under these conditions. Finally, when performing the reaction in dichloromethane (DCM) at 40 °C, a notably higher yield of 98% was obtained after 17 h. Under these conditions, the reaction was highly efficient, yielding a single main product, imidazolidin-2-one **1a**.

The ^1^H NMR analysis of compound **1a**, which is consistent with the literature [27], enabled detailed structural elucidation of the molecular structure. The first fragment, the perhydrobenzimidazole-2-one heterocyclic system, shows a complex signal system corresponding to the hydrogen atoms of the cyclohexane ring, indicating the presence of a saturated structure in this portion like that observed for compound **3a**. The corresponding signal to the *axial* hydrogens (H4_axial_, H5_axial_, H6_axial_, H7_axial_) appears as a multiplet in the 1.22–1.12 ppm range. The hydrogens at the *equatorial* positions (H5_equatorial_, H6_equatorial_) appear in a multiplet in the range of 1.72–1.63 ppm, and the signals for the hydrogens at the H4_equatorial_ and H7_equatorial_ positions also appear as a multiplet in the range of 1.85–1.89 ppm. A key structure fragment is the benzylic methylene, which exhibits two doublet-type signals at 4.36 ppm and 4.48 ppm, with a coupling constant of 15.2 Hz. These signals correspond to the diastereotopic hydrogens, which are coupled to each other. Finally, the aromatic ring shows a multiplet in the range of 7.33–7.23 ppm, with an integration of 10 protons. This indicates the presence of two benzylic rings attached to the methylene benzylic groups. Analysis of compound **1a** by ^13^C NMR revealed key information about the distribution of carbon atoms in the molecular structure. For the perhydrobenzimidazole-2-one system, a signal corresponding to methylene carbon atoms at the C4 and C5 positions of the cyclohexane system is observed at 24.1 ppm. This chemical shift is typical of hydrogen-bonded carbons in a saturated environment, which is consistent with the presence of these carbons in the cyclohexane ring. At 28.4 ppm, methylene carbon atoms are found at the C4 and C7 positions of the cyclohexane system. The slightly higher chemical shift may reflect the proximity of these carbons to chiral centers, which is expected in this portion of the structure. At 61.7 ppm, the signal corresponding to the chiral carbons C3a and C7a is observed. This shift is typical for carbons attached to a nitrogen atom (in this case, within the imidazolidinone ring), and is in the range of chemical shifts expected for these carbon atoms close to a chiral center. At 46.9 ppm, a signal is assigned to a carbon atom corresponding to the methylene benzylic carbon atom. For the aromatic ring, four signals are observed in the range of 137.8–127.2 ppm, which correspond to the carbons of the aromatic system. These chemical shifts are consistent with the presence of a monosubstituted aromatic system. Finally, at 163.6 ppm, the chemical shift corresponding to the carbon of the C=O bond in the imidazolidin-2-one ring is observed.

**Figure 2:**
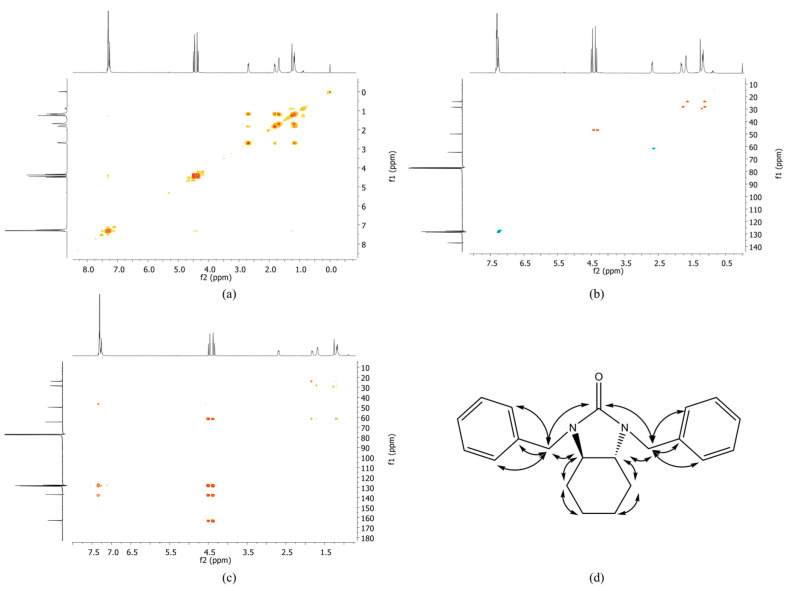
Two-dimensional nuclear magnetic resonance experiments of compound **1a**. (**a**) COSY, (**b**) HMQC, (**c**) HMBC, (**d**) schematic representation of the connectivity between resonances of ^1^H and ^13^C atoms in **1a**.

A detailed analysis of the results obtained from the two-dimensional NMR experiments (Figure 2) performed on compound **1a** is presented below to interpret the interactions and relationships between the protons and carbons present in the proposed structure. The COSY experiment allowed observation of the interactions through bonds between nearby protons, identifying hydrogen groups coupled to each other. In the cyclohexane fragment, the axial protons H4_axial_, H5_axial_, H6_axial_, and H7_axial_ exhibit chemical shifts in the 1.22–1.12 ppm range. These showed correlations with the equatorial protons H5_equatorial_, H6_equatorial_, H4_equatorial_, and H7_equatorial_, which have chemical shifts in the range of 1.72–1.63 ppm and 1.85–1.89 ppm, respectively. The equatorial protons (H5_equatorial_, H6_equatorial_) in the 1.72–1.63 ppm range correlate with the axial protons (H4_axial_, H5_axial_, H6_axial_, H7_axial_) in the 1.22–1.12 ppm range. The H4_equatorial_ and H7_equatorial_ protons (1.85–1.89 ppm) correlate with the axial protons in the chemical shift range of 1.22–1.12 ppm. The protons at the H3a positions (2.75–2.63 ppm) showed correlations with the equatorial protons H4_equatorial_ and H7_equatorial_ and axial protons H4_axial_ and H7_axial_. Regarding the benzylic protons, those at 4.36 ppm showed correlations with the protons at 4.48 ppm, confirming that they are diastereotopic protons. Finally, the protons of the aromatic ring, with displacements in the range of 7.33–7.23 ppm, correlate with each other in the same region.

The HMQC experiment provided information on the direct correlation between the protons and the carbons to which they are attached. The axial protons at positions H4–7_axial_ correlated with two methylene carbons in cyclohexane which appeared as signals at 24.1 and 28.4 ppm. The equatorial protons (1.72–1.63 ppm) correlated with two methylene carbons (24.1 ppm). The protons at positions H4_equatorial_ and H7_equatorial_ (1.85–1.89 ppm) also correlated with the methylene carbons. The protons attached to the chiral center H3a and H7a (2.75–2.63 ppm) correlated with a methine chiral carbon which appeared as a signal at 61.7 ppm, indicating the presence of a chiral center. The diastereotopic benzylic hydrogens at 4.36 and 4.48 ppm correlated with a benzylic carbon at 46.9 ppm. The protons in the 7.33–7.23 ppm region showed correlations with methine-type aromatic carbons in the 127.0–128.4 ppm range. Finally, the HMBC experiment revealed long-distance interactions between the protons and the carbons. The axial protons (1.22–1.12 ppm) correlated with the chiral methine-type carbon (61.7 ppm), suggesting an interaction via long-distance bonds. Like the H_axial_, the equatorial protons (1.72–1.63 ppm) correlated with the chiral methine-type carbon (61.7 ppm). The protons at 2.75–2.63 ppm correlated with the methylene carbons from cyclohexane (24.1 and 28.4 ppm) and a signal for the C=O carbon at 163.6 ppm, suggesting the proximity of this carbonyl group in the structure. The protons at the ArCH_2_N positions (4.36 and 4.48 ppm) correlated with the signal for the C=O carbon (163.6 ppm) and an aromatic carbon at 137.8 ppm. The aromatic ring protons (7.33–7.23 ppm) correlated with methine-type aromatic carbons and the C=O carbon. The results of the COSY, HMQC, and HMBC experiments demonstrated interactions between the protons and carbons of the cyclohexane system, diastereotopic benzylic protons, and the aromatic rings, in addition to the presence of a chiral center and a carbonyl group, confirming the proposed structure of compound **1a**.

With the optimized reaction conditions, the synthesis of imidazoline-2-ones **1b**–**g** was carried out using *p*-substituted aromatic aldehydes. After optimizing the reaction conditions, the desired compounds **1a**–**g** were obtained, which were characterized using ATR-FTIR, HR-MS, ^1^H, and ^13^C NMR. The characterization data are consistent with those described for compound **1a**. ATR-FTIR reveals that in all compounds, the value of the asymmetric CH_2_ stretching band is in a nearby region, between 2929 and 2935 cm^−1^. This value is characteristic of methylene groups (–CH_2_–) attached to a *sp*^3^ carbon atom. The band corresponding to the symmetric CH_2_ stretching shows similar behavior to the asymmetric band, with values between 2851 and 2863 cm^−1^. As in the case of asymmetric stretching, the small variations in frequency between compounds **1a**, **1b**, and **1c** may be influenced by the inductive effects of the benzyloxy and 4-methylphenoxy groups. However, these effects are not large enough to drastically alter the band position. The carbonyl (C=O) stretching frequency is one of the most distinctive bands in the ATR-FTIR spectrum. These bands are located between 1693 and 1700 cm^−1^, consistent with the expected behavior for a C=O attached to an imidazolin-2-one ring. This band’s slight variability suggests that the benzene ring’s substituents may influence the carbonyl group’s interaction with the molecular environment, possibly due to resonance or electronic polarization effects. In the case of compound **1b** (benzyloxy), the band appears at a slightly lower frequency (1698 cm^−1^), which may indicate a slight increase in the electron density over the carbonyl group, affected by the benzyloxy substituent. Finally, the band corresponding to the C–N stretching in the imidazolin-2-one fragment appears in a region close to 1252 cm^−1^ in the case of compound **1a** and in a slightly lower region for the compounds with benzene substituents, **1b** (benzyloxy) and **1c** (4-methylphenoxy), with values of 1239 cm^−1^ and 1249 cm^−1^, respectively. This shift towards lower frequencies could indicate a change in the C-N bond strength due to the influence of the substituent groups, which can induce changes in the electron density over the imidazolin-2-one ring and, therefore, modify the nature of the C-N bond. The ^1^H NMR results show that the signals corresponding to the benzyl hydrogens of the compounds **1a**–**g** are between 3.83 and 4.48 ppm, which reflects variability in the chemical position due to the presence of different substituents in the benzene ring. The specific effects of each substituent are discussed below. For compound **1b**, the signal moves to 4.47 and 4.21 ppm, indicating a slight modification in the electronic environment of the benzene ring due to the presence of the 4-benzyloxy group, which is an electron donor that causes greater electronic density in the benzene ring. A similar effect is observed for compounds **1c**–**1e**, for which the signal of benzyl hydrogens also appears slightly shifted compared to that observed for **1a**. For derivatives with halogen groups, such as **1f**–**g**, the signals are observed at 3.86 and 3.60 ppm, representing a more substantial shift towards a higher field than electron donor compounds. Both bromine and chlorine are electronegative substituents that, through their inductive nature, decrease electronic density in the benzene ring, which could explain the observed shift. Finally, the ^1^C NMR analysis showed resonance signals for benzylic carbons in the range of 46.1–46.9 ppm, with slight variations attributable to the substituent groups on the benzene ring. The carbonyl carbon atoms (C=O) appear as signals between 163.3 and 163.6 ppm, reflecting a constancy in the environment of the carbonyl group that is not significantly altered by the substituents on the ring.

Yields for compounds **1a**–**g** using a multistep protocol are depicted in Table 3. These results showed significant variations in yields depending on the substituents in the starting structure. These results reflect how the substituent’s electronic and steric properties affect both the formation of the diamine and its subsequent cyclization with CDI, impacting the overall yield of the process. Compound **1a** showed the best yields in each stage, with 74% in the diamine production stage and 98% in the cyclization stage, resulting in an overall yield of 73%. The absence of bulky groups or significant electronic effects presumably facilitates the formation of the Schiff base and its subsequent cyclization, highlighting the favorable influence of a system without steric or electronic impediments. Regarding imidazolin-2-one **1b**, introducing the benzyloxy group increased the yield in the diamine production stage (83%), possibly due to electronic interactions of the oxygen group with the carbonyl system of the Schiff base, stabilizing the intermediate. However, the cyclization yield decreased to 84%, which could be attributed to the greater steric complexity of the benzyloxy group, making the reaction with CDI more difficult. The overall yield was 70%, close to that of compound **1a**. For imidazolidin-2-one **1c**, the highest yield was observed in the diamine production stage (90%), suggesting that the 4-methylphenoxy group favors the formation of the Schiff base due to the electron-donating effects of the methyl group in the aromatic ring, which can stabilize the intermediate. However, the cyclization yield was notably lower (63%). Regarding compound **1d**, the bulky and electron-donating effect of the 4-diphenylamine group significantly negatively affected both steps. The cyclization yield was extremely low (22%), indicating that this group severely hinders the formation of the imidazolidin-2-one fragment. The overall yield was the weakest of all the compounds evaluated (17%), reflecting that bulky substituent groups are unfavorable for this type of synthesis. Additional substituents with varying electronic effects were introduced for compounds **1e**, **1f**, and **1g**. Compound **1e**, with the *tert*-butoxy group, showed a relatively low yield in the diamine formation stage (65%) and a modest cyclization yield of 59%. The *tert*-butyl group is an electron-donating group, which affected the aldehyde electrophilicity towards the Schiff base formation and the cyclization stages, reducing yields in both steps. In compound **1f**, introducing the chlorine atom resulted in a yield of 79% in the diamine formation and a high cyclization yield of 95%. Chlorine is an electron-withdrawing group, which would stabilize the electron-rich nitrogen of the Schiff base, facilitating cyclization. The favorable electronic properties of chlorine and relatively low steric hindrance allowed for a high overall yield of 75%, one of the best results among the substituted compounds. Finally, compound **1g**, with a bromo group, exhibited 78% and 94% yields in the diamine and cyclization steps, respectively. The bromine group, like chlorine, is electron-withdrawing, though less strongly so. It also provided favorable stabilization to the Schiff base intermediate, leading to a cyclization yield of 94%. As a result, the overall yield of 73% was like that of **1f**, showing that halogen substitution can significantly improve the cyclization step compared to more sterically demanding or electron-donating groups. These results showed that bulky groups or those with significant electronic effects (**1c**, **1d**, and **1e**) influence the cyclization step.

To improve the yields of compounds **1a**–**g**, conditions for a pseudo-multicomponent synthetic strategy were evaluated, with varying reaction conditions such as CDI stoichiometry, temperature, and reaction time. Moreover, microwave irradiation (MW) was employed in this study. The use of MW irradiation in organic synthesis has proven highly beneficial, particularly in accelerating reactions, increasing yields under milder conditions, and improving product purity [30,31]. The thermal effects of MW irradiation arise from several factors, including the rapid heating rate, localized overheating, formation of “hot spots”, and the selective absorption of radiation by polar compounds. MW irradiation reduces reaction times and increases yields, making the process more efficient compared to conventional methodologies, where heating is slower and less controlled. Furthermore, MW in this type of reaction enabled us to operate under milder conditions, reducing energy consumption.

### 2.2. Pseudo-Multicomponent Experiments for the Synthesis of Compounds ***1a**–**g***

Several experiments were performed to obtain compound **1a**, evaluating the influence of the mentioned variables, to improve the yield of compound **1a**–**g** through a pseudo-multicomponent protocol. Trying to avoid the chemical reaction of reagents such as NaBH_4_ and CDI, THF and DCM were selected as solvents. The final stage of the synthesis was optimized using a 3^3^ full-factorial experimental design, which examined the effects of three key factors—temperature, reaction time, and reactant stoichiometry—each at three levels, as shown in Table S1. The response variable was the reaction yield percentage. The experimental data were analyzed by fitting a quadratic model, which was validated through analysis of variance (ANOVA), which identified significant effects for factors with *p*-values < 0.05 (Table S2). The model’s performance in capturing the variability of the experimental data was assessed using the coefficient of determination (R^2^). Additionally, significant differences in yield percentages among treatments were determined through a post hoc Tukey test.

The analysis of variance (ANOVA) conducted on the experimental data provided valuable insights into the effects of the studied factors and their interactions (Supplementary Materials, Table S2). Initially, the quadratic model was statistically significant, with a *p*-value of 0.0012, confirming its ability to explain the observed variability in the data. The coefficient of determination (R^2^) was 0.7454, indicating that the model explains approximately 74.5% of the variation in the response. As for the main effects, temperature (factor A) significantly impacted reaction yield, with a *p*-value less than 0.0001 and an F-value of 31.90. This indicates that temperature is a key factor influencing the yield. In contrast, reaction time (factor B) yielded a *p*-value of 0.0560, suggesting a marginally significant effect. Although close to the significance threshold, it does not fully meet it, implying that reaction time may influence the yield, but its impact is less pronounced compared to temperature. Conversely, the stoichiometry of the reactants (factor C) was not significant, with a *p*-value of 0.4135, indicating that variations in yield are not attributed to this factor. When examining the interactions between the factors, none of the combinations—temperature and reaction time (interaction AB), temperature and stoichiometry (interaction AC), or reaction time and stoichiometry (interaction BC)—were significant, with *p*-values above 0.05 in all cases. This suggests that no significant interactions between these factors affect the reaction yield. Additionally, the quadratic effects showed that temperature had a significant quadratic influence, with a *p*-value of 0.025, indicating a non-linear relationship between temperature and yield, within the parameter space explored in this experimental design. Finally, the model’s residuals did not display systematic patterns, suggesting that the quadratic model adequately fits the experimental data and does not require further refinement. However, it is important to recognize the empirical nature of this statistical model. Since the design of the experiments enabled construction of a statistical rather than a physicochemical model, the model’s predictions are valid only within the explored experimental space bounded by the upper and lower limits of the factors studied in this work. Extrapolating beyond these boundaries may introduce inaccuracies, requiring additional experimental validation [32]. This limitation is particularly relevant when scaling up the process, as factors not considered in the initial model—such as mass and heat transfer effects—may influence the reaction outcome, necessitating further refinement and optimization.

The results of the Tukey post hoc test, based on the data in Table S3, reveal significant differences in the yields (%) among the different combinations of temperature, time, and stoichiometry. The yields were categorized into distinct groups, with the letter assignments indicating statistical significance. In general, the highest yields were observed under the combination of 40–70 °C and 40–60 min, where treatments T3, T5, T11, and T24 all showed yields over 59% and were grouped with the letter “a” indicating no significant differences between these treatments. The statistical analysis confirmed that temperature had a significant impact on the reaction yield (*p* < 0.0001), while time showed a marginal effect (*p* = 0.0560). In contrast, stoichiometry had no significant impact on the results (*p* = 0.4135), which aligns with the observation that high yields were obtained across different stoichiometric conditions. Conversely, treatments at 100 °C generally exhibited lower yields. For example, T7, T8, and T9, with reaction times of 20, 40, and 60 min, respectively, produced yields ranging from 7% to 14%. These results suggest that excessively high temperatures (100 °C) may be detrimental to the reaction yield, regardless of the reaction time. Finally, a pseudo-multicomponent synthetic protocol was optimized using the discussed statistical analysis to produce compounds **1a**–**d** (Table 4).

Table 4 presents the yields for compounds **1a**–**g** synthesized through two different protocols: a multistep protocol and one-pot protocol. The results suggest that the pseudo-multicomponent protocol is more efficient for synthesizing imidazolidin-2-ones **1a**–**g**, as it offers higher yields in all cases tested. Pseudo-multicomponent reactions (pseudo-MCRs) offer a compelling strategy for synthesizing complex molecules by involving the reactivity of fewer components than traditional multicomponent reactions. These reactions are characterized by having two out of three or more identical components, which, although often seen as undesired in specific synthetic contexts, can provide remarkable efficiency in generating molecular complexity. Pseudo-MCRs play an essential role in synthesizing complex heterocycles, functionalized compounds, and biologically relevant molecules, making them a powerful tool in organic chemistry. Castillo et al. [33] reported a notable example of how pseudo-MCRs can be harnessed effectively. The report discusses aryne-based pseudo-MCRs, where reactive intermediates like arynes mediate reactions to form complex heterocyclic structures. In particular, reactions involving 2-aza-dienes and electron-rich *N*-heteroaryl imines are explored for their ability to synthesize biologically relevant heterocycles such as *N*-arylated 1,2-dihydroisoquinolines. The pseudo-three-component and pseudo-four-component reactions outlined in the study enable the assembly of these heterocycles with high efficiency. A different approach to pseudo-MCRs is reported using Meldrum’s acid, 4-nitrobenzaldehyde, and isocyanides to form intermediates for secondary reactions [34], demonstrating how pseudo-MCRs can be used to produce a range of complex products by manipulating the number of components involved in each step of the reaction. Moreover, steric effects of amidine salts on the formation of *N*-aroylmethylimidazoles and 1*H*-imidazoles by reaction of α-bromoketones with high regioselectivity [35], and the reaction between propargyl amine, an aldehyde, and an electron-deficient alkene [36] to produce highly diastereoselective cycloadducts, exemplify how pseudo-MCRs represent an exciting and efficient strategy in modern synthetic chemistry. This strategy allows the rapid assembly of complex molecules from fewer components, thereby minimizing waste and reducing reaction times, such as our results suggest, showing that the protocol described and studied here corresponds to a new and significant contribution in this class of synthesis strategies.

**Scheme 3:**
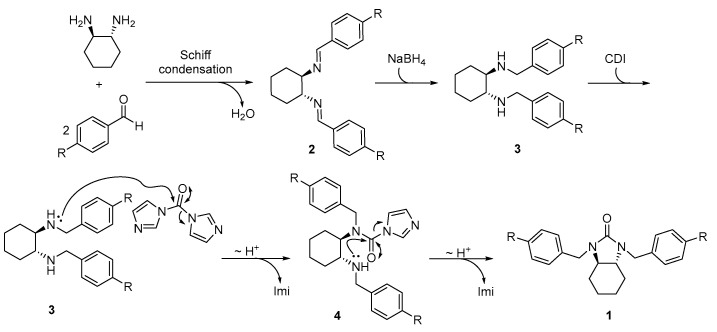
Proposed reaction mechanism for the pseudo-multicomponent synthesis of compounds **1a**–**d**.

Finally, a reaction mechanism is proposed to form compounds **1a**–**g** (Scheme 3). The mechanism begins with the reaction of *trans*-(*R*,*R*)-diaminocyclohexane with two equivalents of the aromatic aldehyde, resulting in a Schiff condensation reaction. In this step, each of the diamine’s amino groups attacks the aldehyde’s carbonyl, generating an intermediate that rapidly rearranges to form a Schiff base **2**, releasing two water molecules. Subsequently, with the addition of a reducing agent, such as sodium borohydride (NaBH_4_), which acts on the Schiff base, each imine group is reduced, resulting in the formation of *N*,*N*-dibenzyldiaminide **3**. This reduction is accompanied by the presence of water in the medium, which facilitates the neutralization of the intermediate, finally forming *N*,*N*-dibenzyldiamine. In the next step, carbodiimide (CDI) is added to the system. CDI acts as an activating agent, initially reacting with one of the amino groups of *N*,*N*-dibenzyldiamine. This attack generates an intermediate *N*-benzyl-*N*-(2-(benzylamino)cyclohexyl)-1*H*-imidazole-1-carboxamide **4** in which the amino group is covalently linked to the carbodiimide. The second amino group of the diamine then attacks this intermediate intramolecularly, forming a cyclic structure. The reaction culminates in the formation of an imidazolin-2-one **1**. The differences in yields are not uniformly large and vary depending on the substituent. In some cases, such as for compound **1d**, the difference is considerably more noticeable, which could indicate that this substituent favors this pseudo-multicomponent protocol pathway.

## 3. Materials and Methods

### 3.1. General Methods and Materials

All reagents and chemicals were purchased commercially (Merck KGaA and/or Sigma-Aldrich, Darmstadt, Germany) and were used without further purification. *Trans*-(*R*,*R*)-diaminocyclohexane was obtained by enantiomeric resolution using L-(+)-tartaric acid and was subsequently liberated using sodium bicarbonate. The progression of the reactions and purifications of the products were monitored by thin layer chromatography (TLC) on silica gel 60 F254 plates (Merck KGaA and/or Sigma-Aldrich, Darmstadt, Germany) under detection at 254 nm. Nuclear magnetic resonance (NMR) experiments were carried out using a Bruker Avance AV-400 MHz spectrometer (Billerica, MA, USA). TMS was used as a reference to provide chemical shifts in δ (ppm). Typical splitting patterns were implemented to define the signal multiplicity (i.e., s, singlet; d, doublet; t, triplet; m, multiplet). The sign of the specific optical rotations for all compounds was determined using a Jasco P-2000 polarimeter (JASCO Co., Ltd., Mary’s Court, PA, USA) in a quartz cell (1.0 cm), and the value reported was the average of 10 measurements. The chemical reactions were carried out in a Single-mode Discover System microwave reactor model 908,005 series DY1030 in a closed vessel, with the temperature controlled between 40 and 100 °C, an internal pressure between 0 and 10 psi, and microwave power of 200 W. ATR-FTIR spectra were recorded on a Jasco FT/IR-6600 type A spectrophotometer (JASCO Co., Ltd., Mary’s Court, PA, USA) attached to an ATR PRO ONE accessory with an incidence angle of 45 degrees and a resolution of 4 cm^−1^. Mass spectrometry was carried out on a UHPLC Shimadzu Nexera X2 chromatograph coupled to a QTOF LCMS9030 spectrometer. Formic acid 0.1%: methanol 40:60 was employed as the mobile phase, with a total run time of 20 min, using a Shim-pack HR-ODS column 150 mm L × 3 mm ID, 80 A°. The analysis was performed in positive and negative ion modes, finding that the most appropriate mode was ESI+.

### 3.2. Multistep Protocol for the Synthesis of Compounds ***1a**–**g***

#### 3.2.1. Synthesis of Compounds **3a**–**g**

Compounds **3a**–**g** were prepared based on the methodology previously reported in the literature, with modifications. *Trans*-(*R*,*R*)-1,2-diamino cyclohexane (1 equiv.) was dissolved in anhydrous methanol (MeOH, 0.3 M), followed by the addition of the respective aldehyde (2.05 equiv.). The mixture was heated to reflux for 1 h under MW irradiation. After cooling to room temperature, sodium borohydride (4 equiv.) was added portion-wise. Once the effervescence subsided, the reaction mixture was heated to reflux for another hour. The reaction was quenched by adding water, followed by rotary evaporation and extraction with dichloromethane (DCM) and water. The crude product was purified based on the nature of the diamines. The compounds were dissolved in DCM, and 1 mL of 37% HCl was added. The mixture was stirred for 5 min and dried over anhydrous sodium sulfate. After solvent evaporation, the resulting diamide salt was washed with ethyl ether.

#### 3.2.2. Synthesis of Compounds **1a**–**g**

The respective diamine **3a**–**g** was dissolved in anhydrous DCM (0.02 M), and carbonyldiimidazole (CDI, 1.0 equiv.) was added. The reaction mixture was heated to reflux overnight and monitored by thin-layer chromatography (TLC). Subsequently, additional CDI (0.2 equiv.) was added, and the mixture was refluxed for 4 h. The reaction was quenched with 0.01 M HCl, followed by extraction. The crude product was purified by column chromatography on silica gel, using petroleum ether/ethyl acetate (5:1) as the eluent to obtain compounds **1a**–**d**. The spectroscopic data for **1a**–**g** are presented in the Supplementary Materials.

### 3.3. Statistical Analysis for the Optimization of the Multicomponent One-Pot Protocol

The final stage of the synthesis of compounds **1a**–**g** was optimized using a 3^3^ full-factorial experimental design. This design assessed the effects of three key factors—temperature, reaction time, and reactant stoichiometry—each at three levels, as shown in Table S1. Experimental runs were randomized to minimize potential systematic biases, and measurements were conducted in triplicate to ensure reliability and accuracy. The response variable was the reaction yield percentage. The analysis of the experimental data was performed by fitting a quadratic model to evaluate the main effects and interactions between the factors, establishing the relationship between the independent variables—temperature, reaction time, and reactant stoichiometry—and the observed response. The model’s validity was assessed through an analysis of variance (ANOVA), where effects with *p*-values < 0.05 were considered significant. The coefficient of determination (R2) was used to evaluate the model’s ability to explain the variability observed in the experimental data. To identify significant differences in the response variable (% yield) between treatments, a post hoc mean comparison was conducted using the Tukey test.

### 3.4. One-Pot Protocol for the Synthesis of Compounds ***1a**–**g***

*Trans*-(*R*,*R*)-1,2-diaminocyclohexane was dissolved in anhydrous THF (0.3 M), followed by the addition of the respective aldehyde (2.05 equiv.). The mixture was heated to reflux for 60 min using microwave irradiation (MW). After cooling to room temperature, sodium borohydride (2.1 equiv.) was added portion-wise. Once the effervescence subsided, the reaction mixture was heated to reflux for 240 min, maintaining MW irradiation. After the diamines **3a**–**g** were formed, carbonyldiimidazole (CDI, 1.1 equiv.) dissolved in anhydrous DCM (0.02 M) was added. The reaction mixture was heated to reflux under microwave irradiation (MW) for 60 min. Subsequently, additional CDI (0.2 equiv.) was added, and the mixture was left overnight. The reaction was quenched with 0.01 M HCl, followed by extraction. The crude product was purified by column chromatography on silica gel, using petroleum ether/ethyl acetate (5:1) as the eluent to obtain compounds **1a**–**g**. The spectroscopic data for **1a**–**g** are presented in the Supplementary Materials.

## 4. Conclusions

The optimization of imidazoline-2-ones **1a**–**g** synthesis through multistep and pseudo-multicomponent one-pot protocols was carried out. A factorial experimental design further optimized the pseudo-multicomponent protocol, identifying temperature as the most critical factor influencing reaction yield, with an optimal range of 40–70 °C. Stoichiometry, in contrast, had no statistically significant effect. The pseudo-multicomponent approach consistently outperformed the traditional multistep method, particularly for sterically hindered compounds like **1d**, where yields increased from 16.9% to 55.4%. This strategy streamlines reaction steps, minimizes by-product formation, and provides a scalable and efficient route to imidazoline-2-ones **1a**–**g**. These results underscore the potential of pseudo-multicomponent reactions as a powerful tool for synthesizing heterocyclic compounds. The developed methodology offers a robust and generalizable approach, balancing efficiency, selectivity, and sustainability.

## Figures and Tables

**Table 1:** Synthesis of diamine **3a** from *trans*-(*R*,*R*)-diaminocyclohexane **3a** with varying solvents and reaction temperatures.

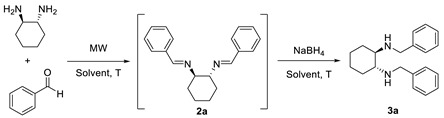						
		Step 1: Schiff Base Formation		Step 2: Reduction to Diamine		
Entry	Solvent	Temperature/°C	Time/min	Temperature/°C	Time/min	Yield/%
1	MeOH	40	80	40	30	74
2	THF	80	80	80	240	74

**Table 2:** Conditions evaluation for the cyclization of **3a** towards compound **1a**.

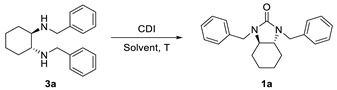				
Entry	Solvent	Temperature/°C	Time/h	Yield/%
1	Toluene	20	64	<5
2	THF	20	64	21
3	DCM	40	17	98

**Table 3:** Synthesis of compounds **1a**–**g** employing a multistep protocol.

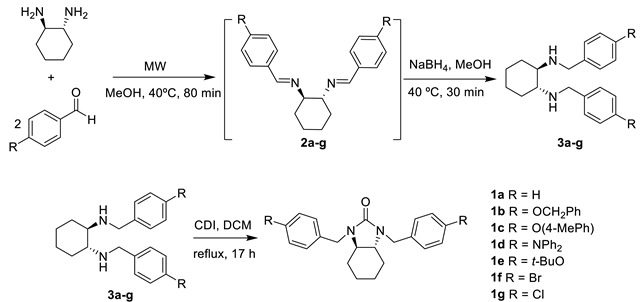					
Entry	Compound	Yield/%	Compound	Yield/%	Overall Yield/%
**1**	**3a**	74	**1a**	98	73
**2**	**3b**	83	**1b**	84	70
**3**	**3c**	90	**1c**	63	57
**4**	**3d**	77	**1d**	22	17
**5**	**3e**	65	**1e**	59	38
**6**	**3f**	79	**1f**	95	75
**7**	**3g**	78	**1g**	94	73

**Table 4:** Synthesis of compounds **1a**–**g** employing pseudo-MCRs and comparison with a multistep protocol.

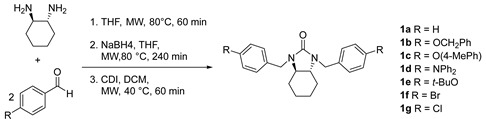			
Entry	Compound	Yield Multistep Protocol/%	Yield Pseudo-Multicomponent Protocol/%
1	**1a**	73	81
2	**1b**	70	76
3	**1c**	57	62
4	**1d**	17	55
5	**1e**	38	58
6	**1f**	75	82
7	**1g**	73	84

## Data Availability

The data that support the findings of this study are available in the Supplementary Materials of this article.

## Supplementary Materials

The following supporting information can be downloaded at: https://www.mdpi.com/article/10.3390/molecules30071415/s1. Figure S1. ATR-FTIR spectra of compounds **3b**–**g**; Figure S2. ATR-FTIR spectra of compounds **1a**–**g**; Figure S3. ^1^H and ^13^C NMR spectra of compound **3a**; Figure S4. ^1^H and ^13^C NMR spectra of compound **3b**; Figure S5. ^1^H and ^13^C NMR spectra of compound **3c**; Figure S6. ^1^H and ^13^C NMR spectra of compound **3d**; Figure S7. ^1^H and ^13^C NMR spectra of compound **3e**; Figure S8. ^1^H NMR spectra of compound **1a**; Figure S9. ^1^H and ^13^C NMR spectra of compound **1b**; Figure S10. ^1^H and ^13^C NMR spectra of compound **1c**; Figure S11. ^1^H and ^13^C NMR spectra of compound **1d**; Figure S12. ^1^H and ^13^C NMR spectra of compound **1e**; Figure S13. ^1^H and ^13^C NMR spectra of compound **1f**; Figure S14. ^1^H and ^13^C NMR spectra of compound **1g**; Figure S15. HRMS spectrum of compound **1a**; Figure S16. HRMS spectrum of compound **1b**; Figure S17. HRMS spectrum of compound **1c**; Figure S18. HRMS spectrum of compound **1d**; Figure S19. HRMS spectrum of compound **1f**; Figure S20. HRMS spectrum of compound **1g**; Table S1. Levels of the factors in the experimental design; Table S2. Analysis of Variance (ANOVA) for the quadratic model evaluating the effects of independent variables on reaction yield; Table S3. Detailed experimental design with observed response and Tukey test results; Table S4. Specific rotation of precursor **3a**–**g** and products **1a**–**g**.
