# Correction: Hoyos-Orozco et al. Synthesis of Imidazolidin-2-ones from *trans*-(*R*,*R*)-Diaminocyclohexane: A Statistical Analysis-Based Pseudo-Multicomponent Protocol. *Molecules* 2025, *30*, 1415

**DOI:** 10.3390/molecules30142890

**Published:** 2025-07-08

**Authors:** Catalina Hoyos-Orozco, Lili Dahiana Becerra, Diego Quiroga

**Affiliations:** Bioorganic Chemistry Laboratory, Facultad de Ciencias Básicas y Aplicadas, Universidad Militar Nueva Granada, Km 2, Cajicá 250247, Colombia; scatalinahoyoso@gmail.com (C.H.-O.); lili.becerra@unimilitar.edu.co (L.D.B.)

## Supplementary Material Correction

In the original publication [1], there was a mistake in Supplementary Material: Figures S15–S20 were missing.

The corrected Figure S15, Figure S16, Figure S17, Figure S18, Figure S19 and Figure S20 appears below. The authors state that the scientific conclusions are unaffected. This correction was approved by the Academic Editor. The original publication has also been updated.

**Figure S15:**
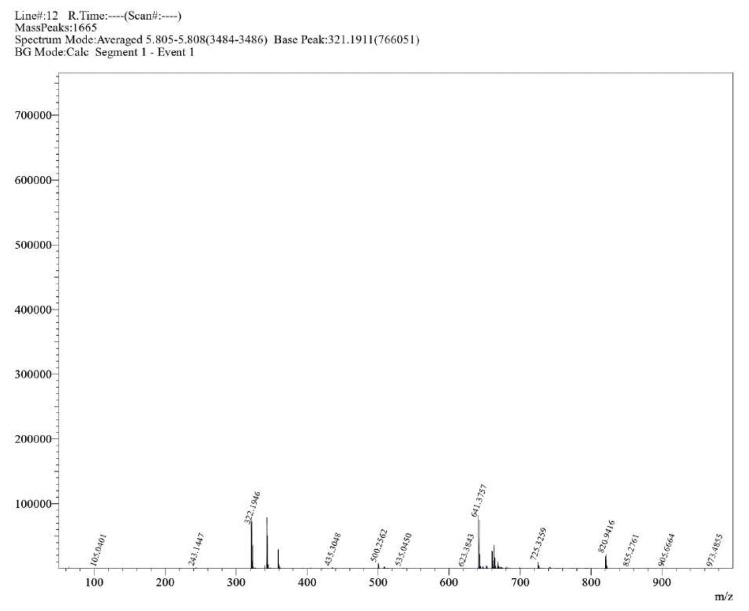
HRMS spectrum of compound **1a**.

**Figure S16:**
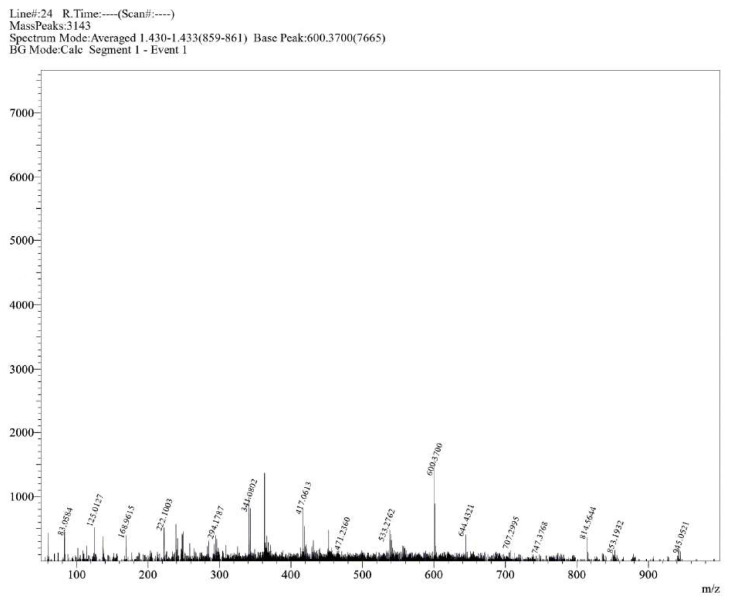
HRMS spectrum of compound **1b**.

**Figure S17:**
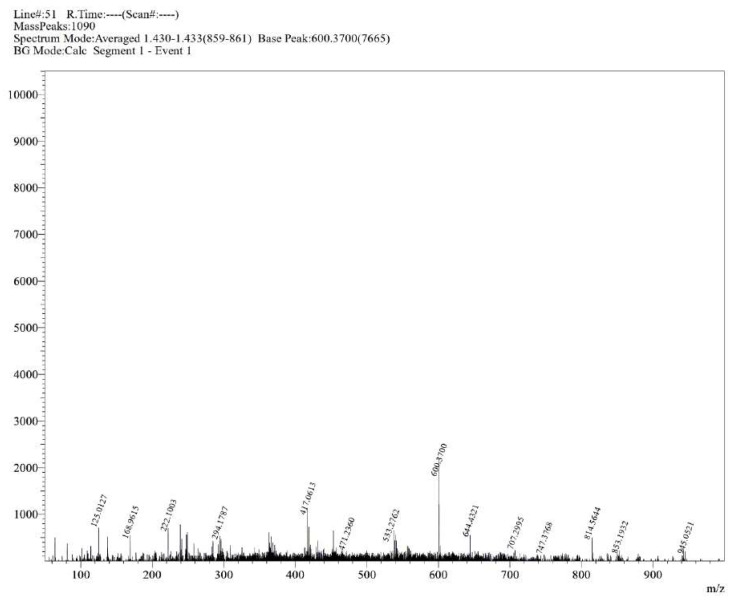
HRMS spectrum of compound **1c**.

**Figure S18:**
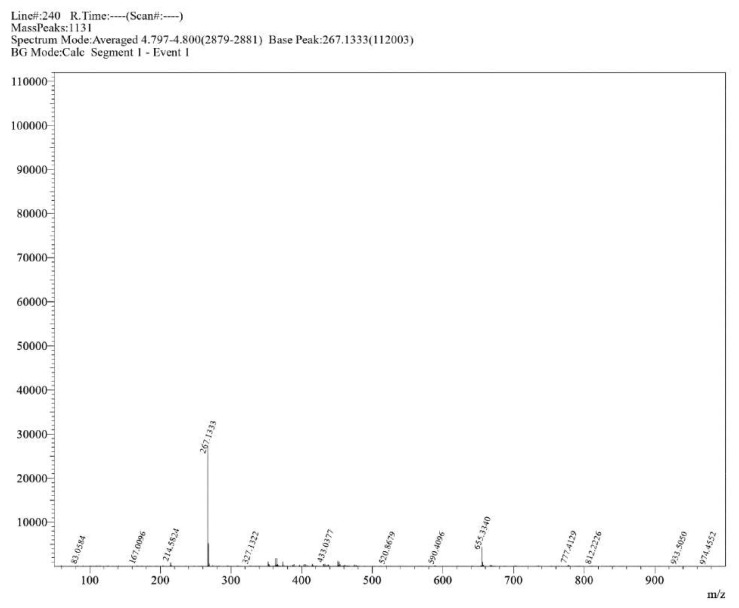
HRMS spectrum of compound **1d**.

**Figure S19:**
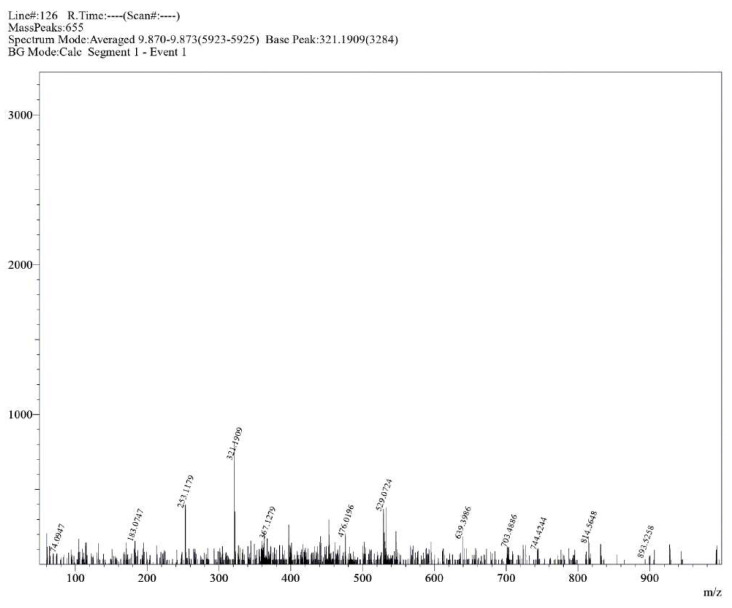
HRMS spectrum of compound **1f**.

**Figure S20:**
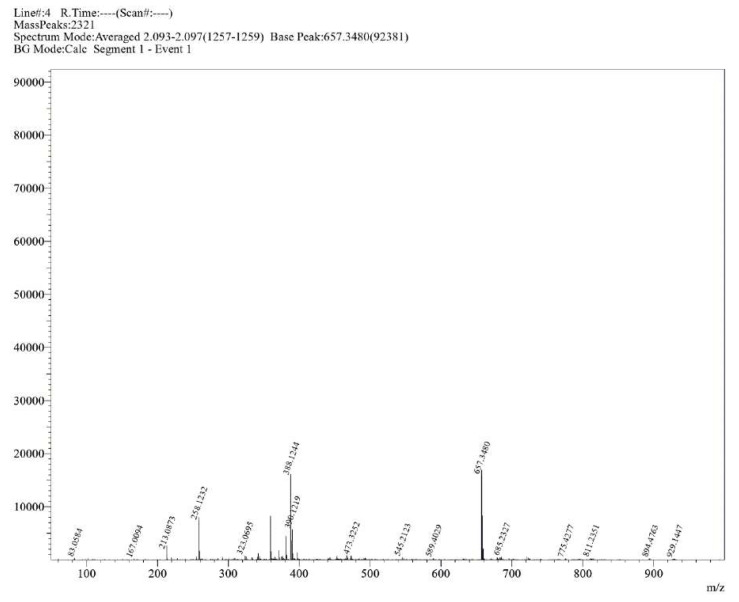
HRMS spectrum of compound **1g**.
