# Supplementary material for: Synthesis of Imidazolidin-2-ones from trans-(R,R)-Diaminocyclohexane: A Statistical Analysis-Based Pseudo-Multicomponent Protocol
Source: Molecules. 2025 Mar 22;30(7):1415. doi: 10.3390/molecules30071415 (PMC11990649; doi:10.3390/molecules30071415)
Supplement: Supplementary file 1 [file molecules-30-01415-s001.zip › molecules-3479845-supplementary.pdf]

Supporting Information

Synthesis of imidazolidin-2-ones from *trans*-(*R*,*R*)-diaminocyclohexane: an statistical analysis-based one-pot protocol

Catalina Hoyos-Orozco, Lili Dahiana Becerra, and Diego Quiroga

Content

**Content**	**Page**
Characterization data of compounds **3a-g** and **1a-g**	2
**Figure S1.** ATR-FTIR spectra of compounds **3b-g**	7
**Figure S2.** ATR-FTIR spectra of compounds **1a-g**	7
**Figure S3.** ^{1}H and ^{13}C NMR spectra of compound **3a**	8
**Figure S4.** ^{1}H and ^{13}C NMR spectra of compound **3b**	9
**Figure S5.** ^{1}H and ^{13}C NMR spectra of compound **3c**	10
**Figure S6.** ^{1}H and ^{13}C NMR spectra of compound **3d**	11
**Figure S7.** ^{1}H and ^{13}C NMR spectra of compound **3e**	12
**Figure S8.** ^{1}H NMR spectra of compound **1a**	13
**Figure S9.** ^{1}H and ^{13}C NMR spectra of compound **1b**	14
**Figure S10.** ^{1}H and ^{13}C NMR spectra of compound **1c**	15
**Figure S11.** ^{1}H and ^{13}C NMR spectra of compound **1d**	16
**Figure S12.** ^{1}H and ^{13}C NMR spectra of compound **1e**	17
**Figure S13.** ^{1}H and ^{13}C NMR spectra of compound **1f**	18
**Figure S14.** ^{1}H and ^{13}C NMR spectra of compound **1g**	19
**Figure S15.** HRMS spectrum of compound **1a**	20
**Figure S16.** HRMS spectrum of compound **1b**	21
**Figure S17.** HRMS spectrum of compound **1c**	22
**Figure S18.** HRMS spectrum of compound **1d**	23
**Figure S19.** HRMS spectrum of compound **1f**	24
**Figure S20.** HRMS spectrum of compound **1g**	25
**Table S1**. Levels of the factors in the experimental design.	26
**Table S2**. Analysis of Variance (ANOVA) for the Quadratic Model Evaluating the Effects of Independent Variables on Reaction Yield	27
**Table S3.** Detailed Experimental Design with Observed Response and Tukey Test Results	28
**Table S4.** Specific rotation of precursor **3a-g** and products **1a-g**	29

(1*R*,2*R*)-*N*[1],*N*[2]-dibenzylcyclohexane-1,2-diamine 3a

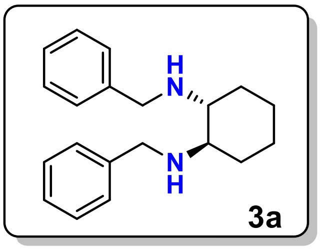

Colorless wax. ^{1}H NMR (400 MHz, $CDCl_3$) δ 7.37 – 7.22 (*m*, 10H), 3.91 (*d*, *J* = 13.1 Hz, 2H), 3.66 (*d*, *J* = 13.1 Hz, 2H), 2.32 – 2.27 (*m*, 2H), 2.20 – 2.12 (*m*, 2H), 1.75 – 1.70 (*m*, 2H), 1.27 – 1.18 (*m*, 2H), 1.14 – 1.02 (*m*, 2H).^{13}C NMR (101 MHz, $CDCl_3$) δ 140.2, 128.4, 128.1, 126.9, 60.5, 50.5, 31.1, 24.9. $[\alpha]_D^{25} = -38.01\,° \pm 0.01$ (c 0.50 $MeOH$). Characterization data are consistent with the reported by Sharma, M.; Joshi, P.; Kumar, N.; Joshi, S.; Rohilla, R. K.; Roy, N.; Rawat, D. S., *Eur. J. Med. Chem.* 2011, 46, 480-487.

(1*R*,2*R*)-*N*[1],*N*[2]-bis(4-(benzyloxy)benzyl)cyclohexane-1,2-diaminium chloride 3b

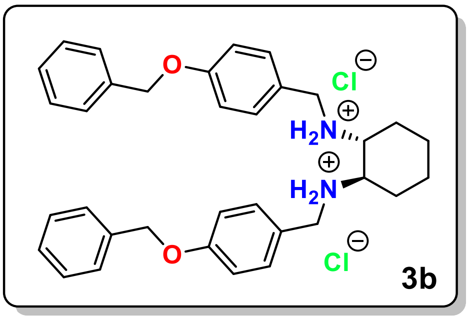

White solid. M.P: 176 – 178 ºC (from DCM), ^{1}H NMR (400 MHz, $CDCl_3$) δ 10.28 (*s*, 2H), 8.96 (*s*, 2H), 7.63 (*d*, *J* = 8.2 Hz, 4H), 7.34 – 7.20 (*m*, 10H), 6.86 (*d*, *J* = 8.3 Hz, 4H), 4.85 (*s*, 4H), 3.95 (*d*, *J* = 12.2 Hz, 2H), 3.70 (*d*, *J* = 12.2 Hz, 2H), 3.55 – 3.46 (*m*, 2H), 2.07 – 2.00 (*m*, 4H), 1.85 – 1.76 (*m*, 2H), 1.33 – 1.22 (*m*, 2H). ^{13}C NMR (101 MHz, $CDCl_3$) δ 159.4, 136.2, 132.9, 128.6, 128.2, 127.5, 122.5, 114.7, 70.0, 57.5, 48.3, 26.8, 22.9. IR (neat, ATR)(cm-1): 2951, 2870, 2686, 2648, 1606, 1513, 1243, 1180. $[\alpha]_D^{25} = -23.71\,° \pm 0.01$ (c 0.01 DCM).

(1*R*,2*R*)-*N*[1],*N*[2]-bis(4-(*p*-tolyloxy)benzyl)cyclohexane-1,2-diaminium chloride 3c

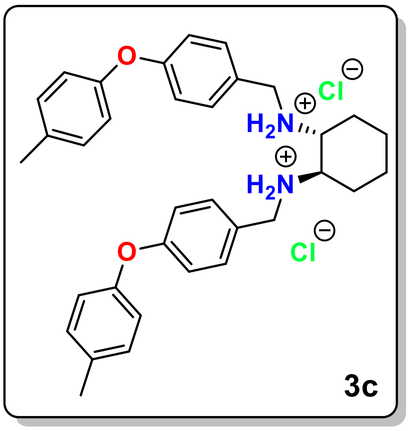

White solid. M.P: 184-186ºC (from DCM).^{1}H NMR (400 MHz, $CDCl_3$) δ 10.15 (*s*, 2H), 8.82 (*s*, 2H), 7.67 (*d*, *J* = 8.2 Hz, 4H), 7.05 (*d*, *J* = 8.2 Hz, 4H), 6.73 – 6.68 (*m*, 8H), 3.93 (*d*, *J* = 11.9 Hz, 2H), 3.62 (*d*, *J* = 11.9 Hz, 2H), 3.34 – 3.27 (*m*, 2H), 2.28 (*s*, 6H), 2.11 – 2.08 (*m*, 2H), 1.84 – 1.65 (*m*, 2H), 1.30 – 1.20 (*m*, 4H). ^{13}C NMR (101 MHz, $CDCl_3$) δ 158.7, 153.3, 133.8, 133.4, 130.4, 124.3, 119.5, 117.2, 57.6, 48.1, 26.3, 22.8, 20.7. IR (neat, ATR)(cm-1): 2948, 2871, 2679, 2643, 1599,1498, 1238, 816. $[\alpha]_D^{25} = -23.93\,° \pm 0.01$ (c 0.01 DCM).

(1*R*,2*R*)-*N*[1],*N*[2]-bis(4-(diphenylamino)benzyl)cyclohexane-1,2-diamine 3d

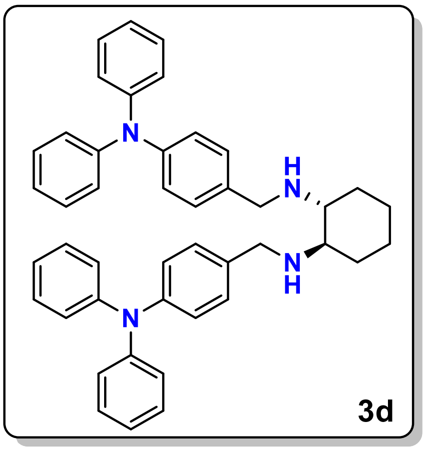

3d

Yellow resin. ^{1}H NMR (400 MHz, $CDCl_3$) δ 7.33 – 7.12 (*m*, 8H), 7.09 – 6.93 (m, 20H), 3.93 (*d*, *J* = 12.8 Hz, 2H), 3.68 (*d*, *J* = 12.7 Hz, 2H), 2.25 – 2.12 (*m*, 2H), 1.78 – 1.66 (*m*, 4H), 1.26 (*m*, 4H). ^{13}C NMR (101 MHz, $CDCl_3$) 147.8, 147.2, 129.5, 129.3, 129.3, 129.3, 124.3, 124.1, 122.9, 60.4, 55.0, 50.1, 30.8, 24.87. IR (neat, ATR)(cm-1): 2929, 2847, 1588, 1486, 1276, 754. $[\alpha]_D^{25} = -13.14° \pm 0.01$ (c 0.01 DCM).

(1*R*,2*R*)-*N*[1],*N*[2]-bis(4-(*tert*-butoxy)benzyl)cyclohexane-1,2-diamine 3e

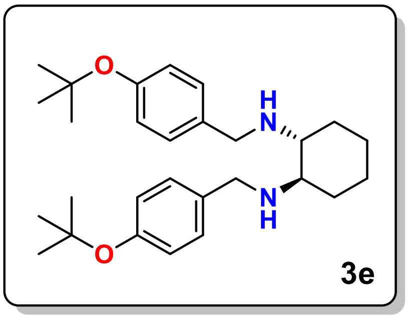

3e

Yellow wax, yield. ^{1}H NMR (400 MHz, $CDCl_3$) δ 7.19 (*d*, *J* = 8.4 Hz, 4H), 6.91 (*d*, *J* = 8.4 Hz, 4H), 3.84 (*d*, *J* = 12.9 Hz, 2H), 3.58 (*d*, *J* = 12.9 Hz, 2H), 2.25 – 2.12 (*m*, 2H), 1.78 – 1.66 (*m*, 4H), 1.30 – 1.24 (*m*, 4H), 1.32 (*s*, 18H). ^{13}C NMR (101 MHz, $CDCl_3$) δ 154.2, 135.7, 128.6, 124.2, 78.4, 60.9, 50.4, 31.5, 28.9, 25.1. IR (neat, ATR)(cm-1): 2970, 2922, 1502, 1362, 1164, 898. $[\alpha]_D^{25} = -35.81° \pm 0.01$ (c 0.01 DCM).

(1*R*,2*R*)-*N*[1],*N*[2]-bis(4-(bromo)benzyl)cyclohexane-1,2-diamine 3f

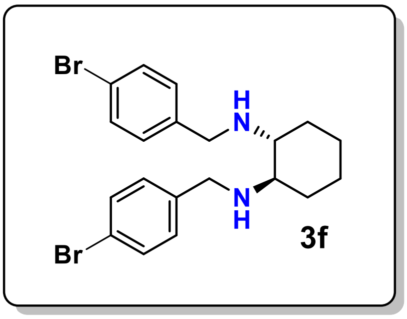

3f

Yellow wax. ^{1}H NMR (400 MHz, DMSO-*d6*): 9.96 (*s*, 2H), 7.68–7.58 (*m*, 8H), 4.29 (*d*, *J* = 15 Hz, 2H), 4.12 (*d*, *J* = 15 Hz, 2H), 3.57 (*s*, 2H), 2.30–2.26 (*m*, 2H), 1.85–1.73 (*m*, 4H), 1.18–1.13 (*s*, 2H). ^{13}C NMR (101 MHz, DMSO-*d6*): 133.7, 133.6, 132.2, 131.8, 123.2, 60.6, 57.0, 49.0, 47.7, 26.5, 23.1, 21.6, 14.9. IR (neat, ATR) (cm−1): 2924, 2854, 2630, 1594, 1543, 1488, 1377, 1072, 1013, 808. $[\alpha]_D^{25} = -12.34° \pm 0.02$ (c 0.01 MeOH). Characterization data are consistent with the reported by Sharma, M.; Joshi, P.; Kumar, N.; Joshi, S.; Rohilla, R. K.; Roy, N.; Rawat, D. S., *Eur. J. Med. Chem.* 2011, 46, 480-487.

(1*R*,2*R*)-*N*[1],*N*[2]-bis(4-(chloro)benzyl)cyclohexane-1,2-diamine 3g

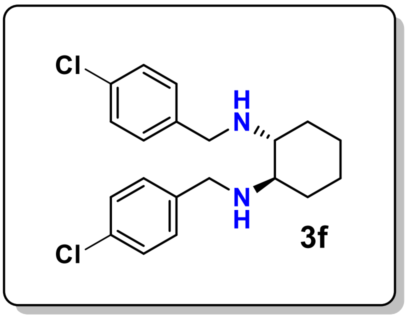

Yellow wax. ^{1}H NMR (400 MHz, DMSO-d6): 7.66 (*d*, *J* = 6 Hz, 4H), 7.45 (*d*, *J* = 6 Hz, 4H), 4.28 (*d*, *J* = 12 Hz, 2H), 4.12 (*d*, *J* = 12 Hz, 2H), 3.74 (*s*, 2H), 2.25–2.29 (*m*, 2H), 1.72 (*m*, 4H), 1.19 (*s*, 2H). IR (neat, ATR) (cm−1): 2938, 2706, 1600, 1494, 1457, 1139, 1095, 1017, 814, 620. $[\alpha]_D^{25} = -14.11\,° \pm 0.01$ (c 0.01 MeOH). Characterization data are consistent with the reported by Sharma, M.; Joshi, P.; Kumar, N.; Joshi, S.; Rohilla, R. K.; Roy, N.; Rawat, D. S., *Eur. J. Med. Chem.* 2011, 46, 480-487.

(3a*R*,7a*R*)-1,3-dibenzyloctahydro-2*H*-benzo[*d*]imidazol-2-one 1a

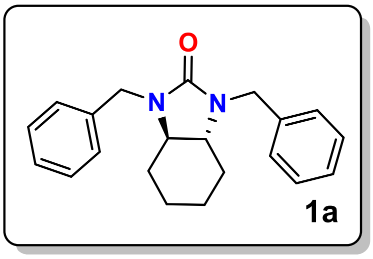

Colorless wax.^{1}H NMR (400 MHz, $CDCl_3$) δ 7.33 – 7.23 (*m*, 10H), 4.48 (*d*, *J* = 15.2 Hz, 2H), 4.36 (*d*, *J* = 15.2 Hz, 2H), 2.75 – 2.63 (*m*, 2H), 1.85 – 1.89 (*m*, 2H), 1.72 – 1.63 (*m*, 2H), 1.22 – 1.12 (*m*, 4H). ^{13}C NMR (101 MHz, $CDCl_3$) 163.6, 137.8, 128.4, 128.3, 127.2, 61.7, 46.9, 28.4, 24.1. IR (neat, ATR)(cm^{-1}): 2933, 2857, 1697, 1252, 700. $[\alpha]_D^{25} = +8.21\,° \pm 0.01$ (c 0.01 DCM). ESI+-HRMS m/z [M+H]$^+$, 322.1946 (calcd. 321.1967, $C_{21}H_{25}N_2O$). Characterization data are consistent with the reported by Prusinowska, N.; Szymkowiak, J.; Kwit, M., *J. Org. Chem*. 2023, 88, 285-299.

(3a*R*,7a*R*)-1,3-bis(4-(benzyloxy)benzyl)octahydro-2*H*-benzo[*d*]imidazol-2-one 1b

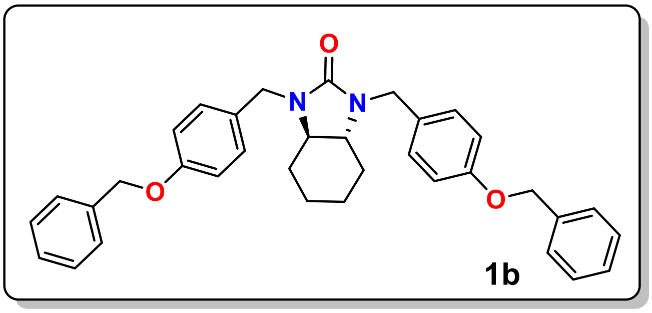

White solid. M.P 131ºC (DCM), ^{1}H NMR (400 MHz, $CDCl_3$) δ 7.45 – 7.30 (*m*, 10H), 7.24 – 7.17 (*m*, 4H), 6.95 – 6.87 (*m*, 4H), 5.04 (*s*, 4H), 4.47 (*d*, *J* = 15.0 Hz, 2H), 4.21 (*d*, *J* = 15.0 Hz, 2H), 2.68 – 2.60 (*m*, 2H), 1.88 – 1.8º (*m*, 2H), 1.72 – 1.67 (*m*, 2H), 1.22 – 1.12 (*m*, 4H). ^{13}C NMR (101 MHz, $CDCl_3$) δ 163.5, 157.9, 136.9, 130.0, 129.5, 128.5, 127.9, 127.5, 114.6, 69.9, 61.2, 46.1, 28.2, 24.1. IR (neat, ATR)(cm-1): 2929, 2858, 1698, 1509, 1239, 735. $[\alpha]_D^{25} = +0.75\,° \pm 0.01$ (c 0.01 DCM). ESI+-HRMS m/z [M+H]$^+$, 533.2762 (calcd. 533.2804, $C_{35}H_{37}N_2O_3$).

(3a*R*,7a*R*)-1,3-bis(3-(*p*-tolyloxy)benzyl)octahydro-2*H*-benzo[*d*]imidazol-2-one 1c

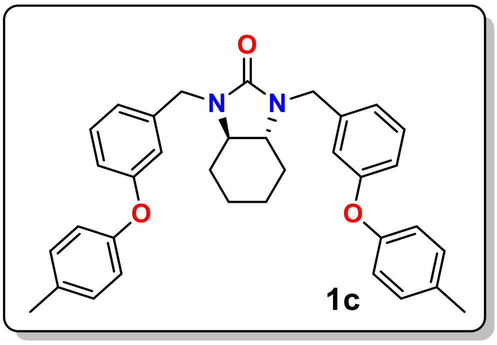

Colorless wax.^{1}H NMR (400 MHz, $CDCl_3$) δ 7.26 – 7.21 (*m*, 2H), 7.15 – 7.10 (*m*, 4H), 7.04 – 7.00 (*m*, 2H), 6.93 – 6.82 (*m*, 8H), 4.36 (*s*, 4H), 2.73 – 2.66 (*m*, 2H), 2.33 (*s*, 6H), 1.80 – 1.76 (*m*, 3H), 1.73 – 1.68 (*m*, 3H), 1.21 – 1.14 (*m*, 4H). ^{13}C NMR (101 MHz, CDCl3) δ = 163.3, 157.7, 154.6, 139.8, 132.8, 130.2, 129.7, 122.6, 118.9, 118.1, 117.0, 61.8, 46.7, 28.4, 24.1, 20.7. IR (neat, ATR)(cm-1): 2929, 2851, 1700, 1504, 1249, 1207, 824, 765. $[\alpha]_D^{25} = +5.49\,° \pm\ 0.01\ (c\ 0.01\ DCM)$. ESI+-HRMS m/z $[M+H]^+$, 533.2762 (calcd. 533.2804, $C_{35}H_{37}N_2O_3$).

(3a*R*,7a*R*)-1,3-bis(4-(diphenylamino)benzyl)octahydro-2*H*-benzo[*d*]imidazol-2-one 1d

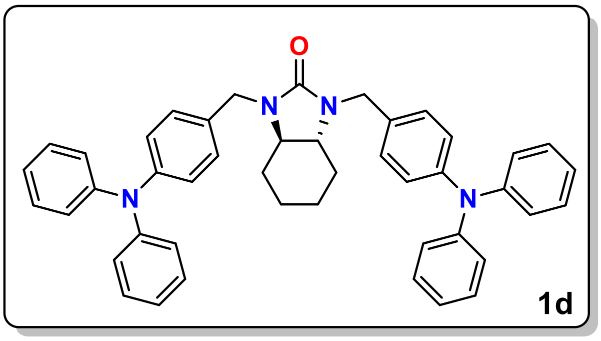

Yellow wax. ^{1}H NMR (400 MHz, $CDCl_3$) δ 7.23 (*dd*, *J* = 8.5, 7.3 Hz, 7H), 7.16 (*d*, *J* = 8.5 Hz, 4H), 7.07 (*dt*, *J* = 7.6, 1.2 Hz, 9H), 7.03 – 6.98 (*m*, 10H), 4.41 (*d*, *J* = 15.2 Hz, 2H), 4.29 (*d*, *J* = 15.2 Hz, 2H), 2.76 (*s*, 2H), 1.91 (*d*, *J* = 8.7 Hz, 3H), 1.77 (*s*, 2H), 1.25 (*s*, 4H). ^{13}C NMR (101 MHz, $CDCl_3$) δ 163.5, 147.7, 146.8, 131.8, 129.2, 129.1, 129.0, 124.1, 123.8, 122.6, 61.6, 46.3, 28.3, 24.2**.** IR (neat, ATR)(cm-1): 2930, 2852, 1670, 1504, 1231, 1210. $[\alpha]_D^{25} = +13.14° \pm\ 0.01\ (c\ 0.01\ DCM)$. ESI+-HRMS m/z $[M+H]^+$, 655.3340 (calcd. 655.3437, $C_{45}H_{43}N_4O$).

(3a*R*,7a*R*)-1,3-bis(4-(*t*ert-butoxy)benzyl)octahydro-2*H*-benzo[*d*]imidazol-2-one 1e

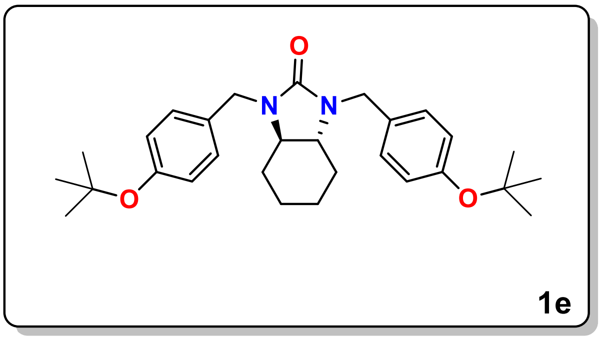

Yellow wax. ^{1}H NMR (400 MHz, $CDCl_3$) δ 7.16 (*d*, *J* = 8.5 Hz, 4H), 6.92 (*d*, *J* = 8.5 Hz, 4H), 4.40 (*d*, *J* = 15.2 Hz, 2H), 4.24 (*d*, *J* = 15.2 Hz, 2H), 2.81 – 2.73 (*m*, 2H), 1.80 – 1.74 (*m*, 4H), 1.47 – 1.38 (*m*, 4H), 1.33 (*s*, 18H). ^{13}C NMR (101 MHz, $CDCl_3$) δ 164.4, 154.6, 132.5, 128.8, 124.3, 78.6, 59.2, 45.7, 29.0, 24.1**.** IR (neat, ATR)(cm-1): 2941, 2855, 1696, 1507, 1246, 1209. $[\alpha]_D^{25} = +60.45° \pm\ 0.01\ (c\ 0.01\ DCM)$.

(3a*R*,7a*R*)-1,3-bis(4-bromobenzyl)octahydro-2*H*-benzo[*d*]imidazol-2-one 1f

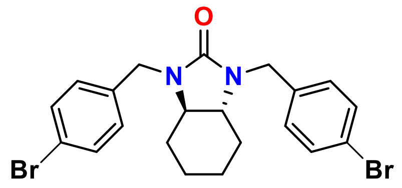

1f

Yellow wax. ^{1}H NMR (400 MHz, $CDCl_3$) δ 7.33 – 7.20 (*m*, 8H), 3.85 (*d*, *J* = 13.4 Hz, 2H), 3.61 (*d*, *J* = 13.4 Hz, 2H), 2.30 – 2.13 (*m*, 2H), 1.79 – 1.66 (m, 4H), 1.33 – 1.04 (m, 4H). ^{13}C NMR (101 MHz, $CDCl_3$) δ 164.2, 139.4, 132.5, 129.4, 128.5, 60.8, 50.1, 31.5, 25.0. IR (neat, ATR)(cm-1): 2951, 2849, 1689, 1509, 1240, 1213. $[\alpha]_D^{25} = +44.25° \pm 0.02\ (c\ 0.01\ DCM)$. ESI+-HRMS m/z [M+H]$^+$, 476.0196 (calcd. 476.0099, $C_{21}H_{22}Br_2N_2O$).

(3a*R*,7a*R*)-1,3-bis(4-chlorobenzyl)octahydro-2*H*-benzo[*d*]imidazol-2-one 1g

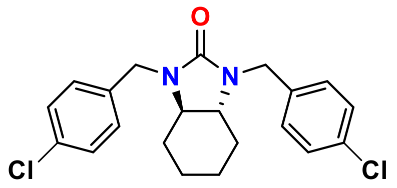

1g

Yellow wax. ^{1}H NMR (400 MHz, $CDCl_3$) δ 7.23 (*dd*, *J* = 8.5, 7.3 Hz, 7H), 7.16 (*d*, *J* = 8.5 Hz, 4H), 7.07 (*dt*, *J* = 7.6, 1.2 Hz, 9H), 7.03 – 6.98 (*m*, 10H), 4.41 (*d*, *J* = 15.2 Hz, 2H), 4.29 (*d*, *J* = 15.2 Hz, 2H), 2.76 (*s*, 2H), 1.91 (*d*, *J* = 8.7 Hz, 3H), 1.77 (*s*, 2H), 1.25 (*s*, 4H). ^{13}C NMR (101 MHz, $CDCl_3$) δ 164.2, 140.0, 136.8, 131.4, 129.8, 60.8, 50.2, 31.5, 25.0. IR (neat, ATR)(cm-1): 2945, 2852, 1689, 1501, 1227, 1215. $[\alpha]_D^{25} = +31.17° \pm 0.01\ (c\ 0.01\ DCM)$. ESI+-HRMS m/z [M]$^+$, 388.1244 (calcd. 388.1103, $C_{21}H_{22}Cl_2N_2O$); [M+2]$^+$, 390.1219 (calcd. 390.1074, $C_{21}H_{22}{}^{35}Cl^{37}ClN_2O$).

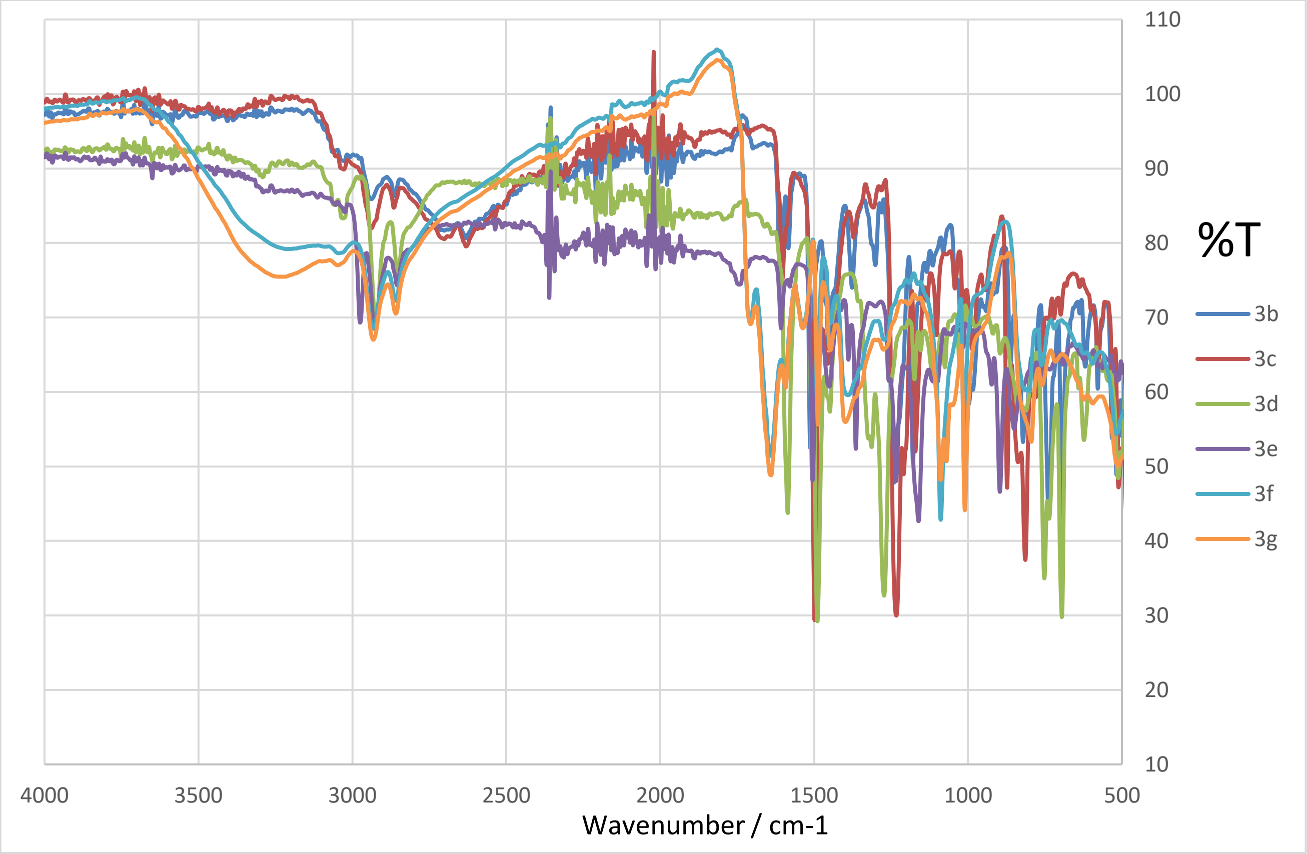

Figure S1. ATR-FTIR spectra of compounds **3b-g**

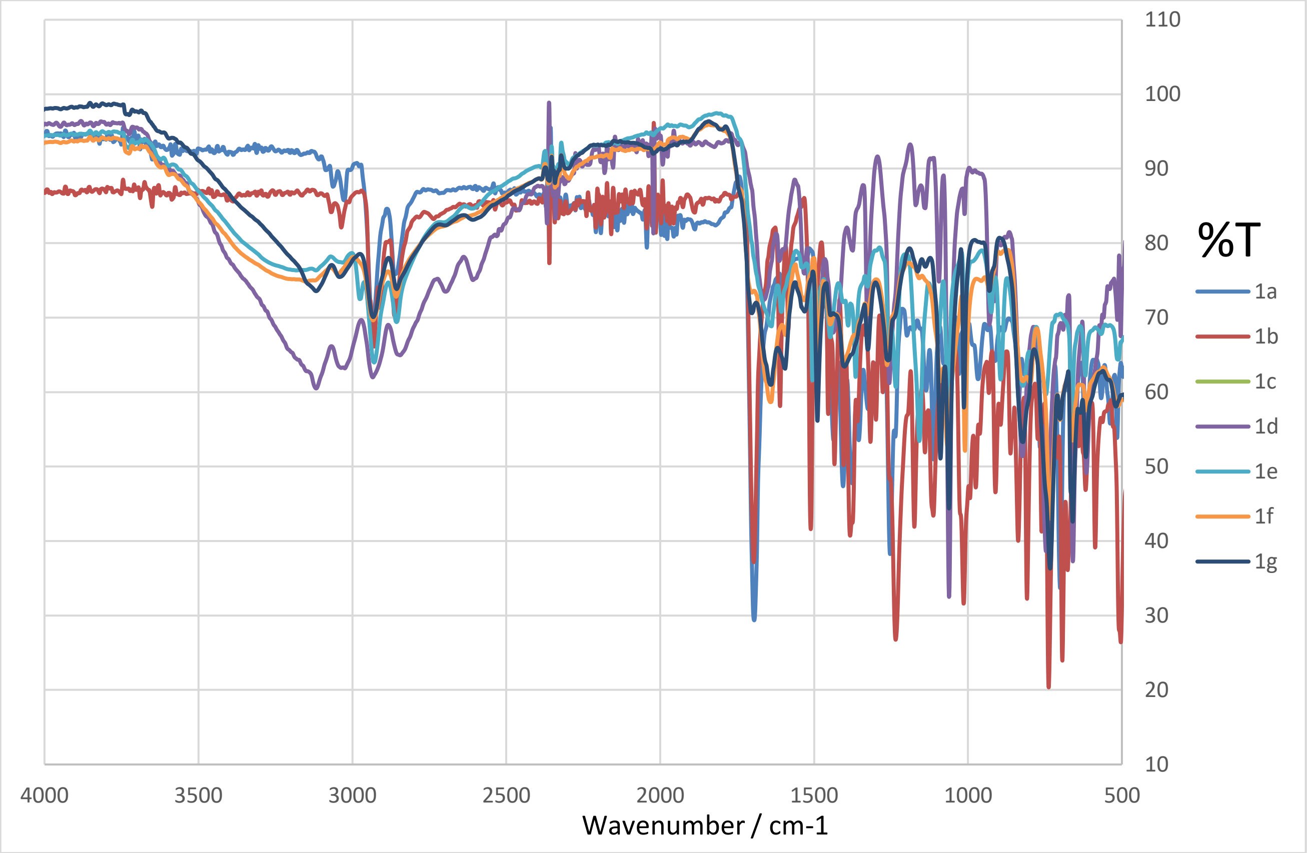

Figure S2. ATR-FTIR spectra of compounds **1a-g**

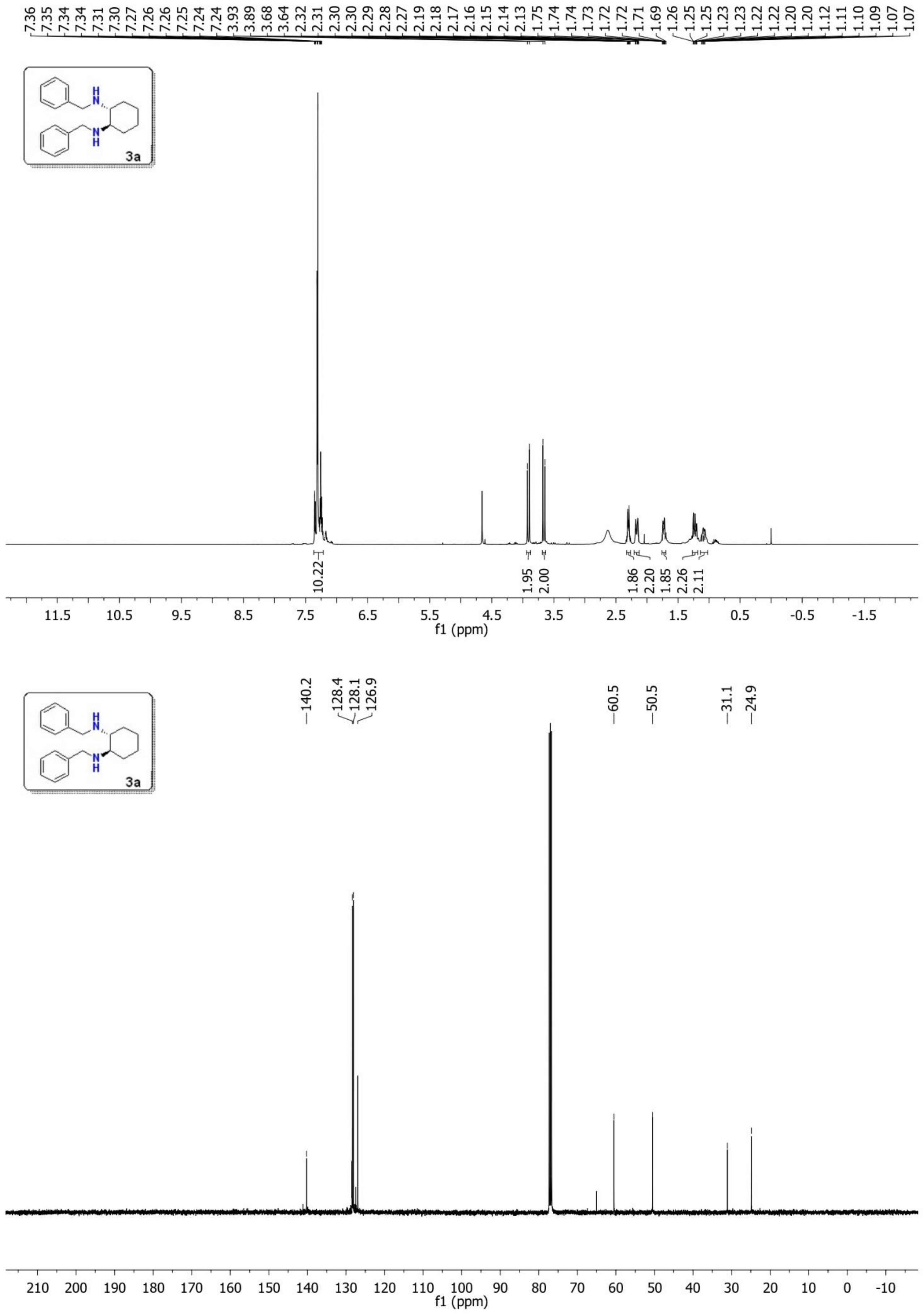

Figure S3. ^{1}H and ^{13}C NMR spectra of compound **3a**

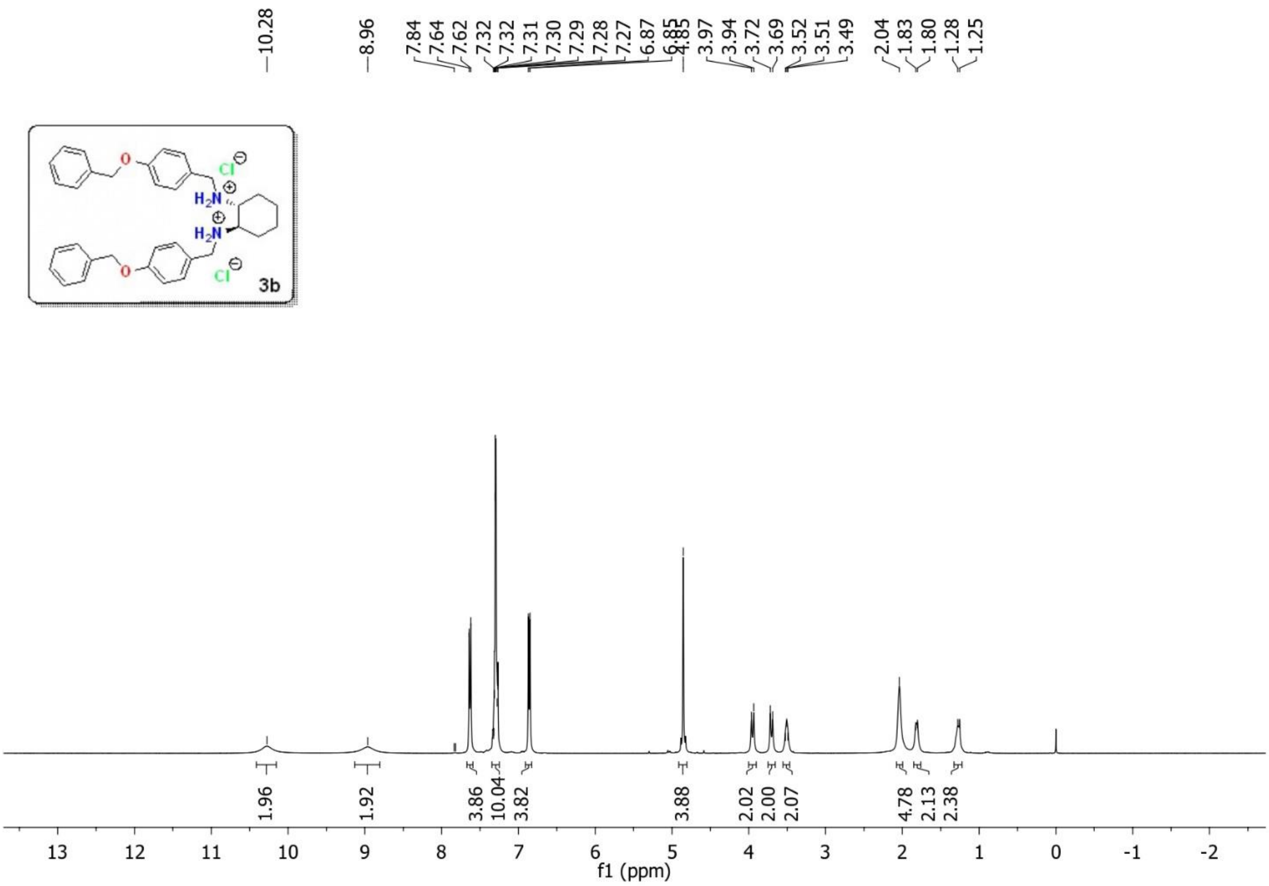

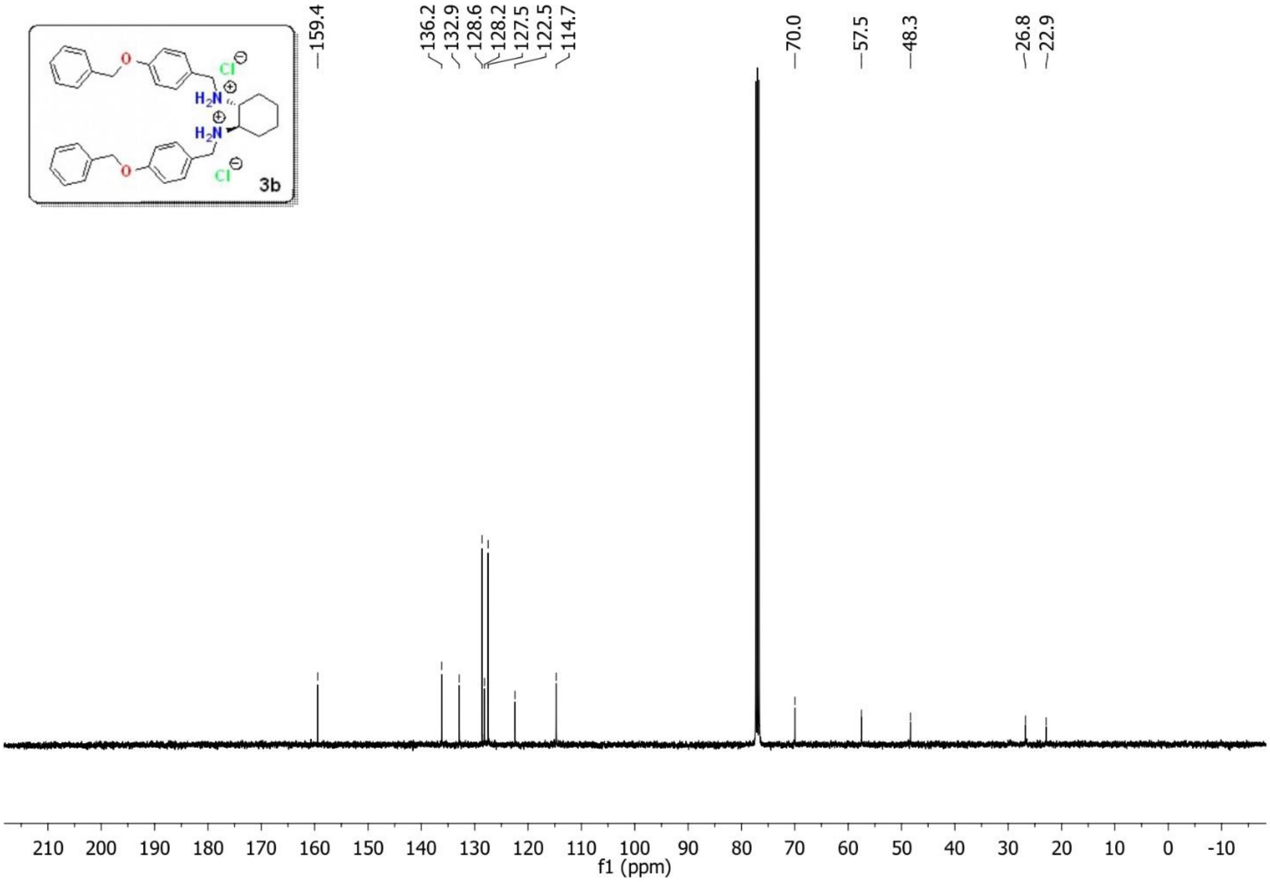

Figure S4. ^{1}H and ^{13}C NMR spectra of compound 3b

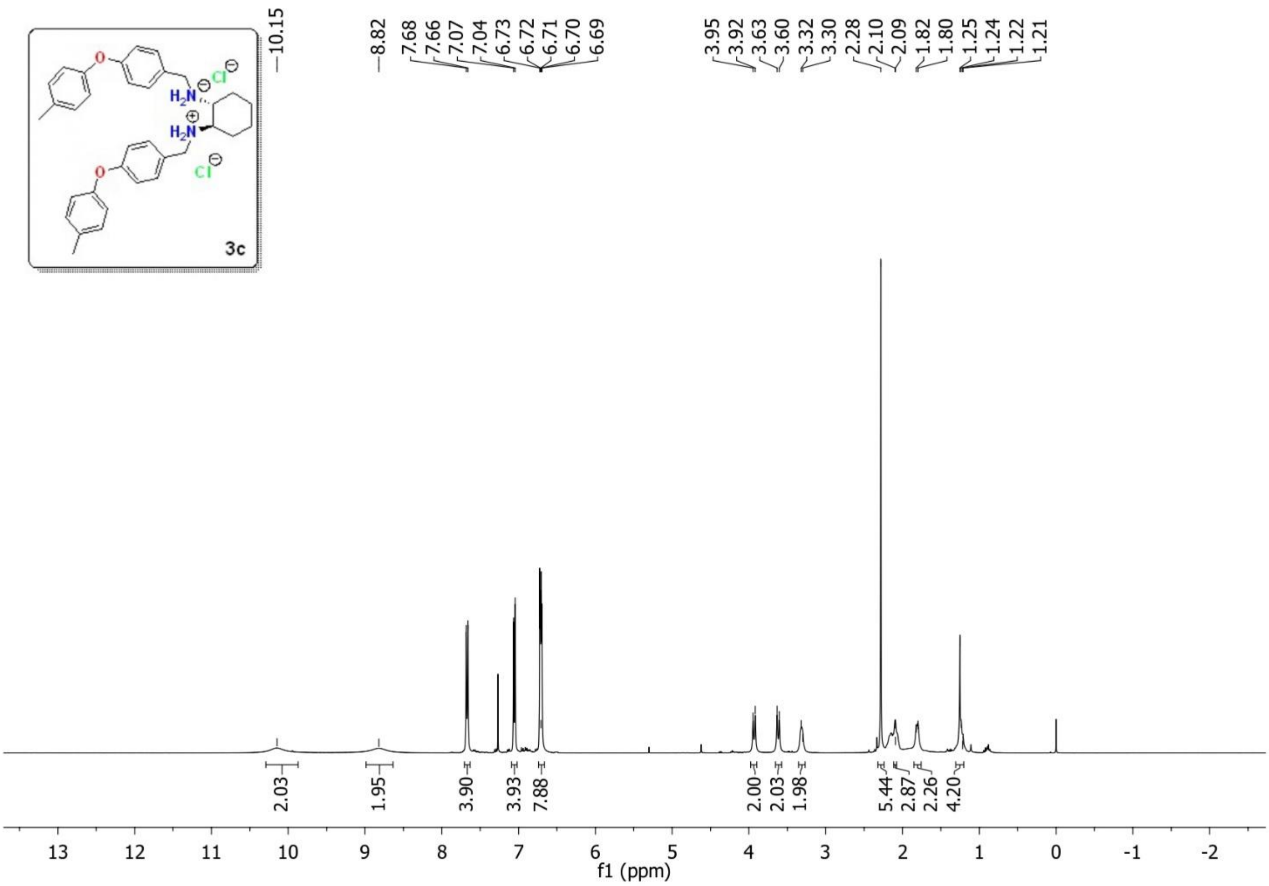

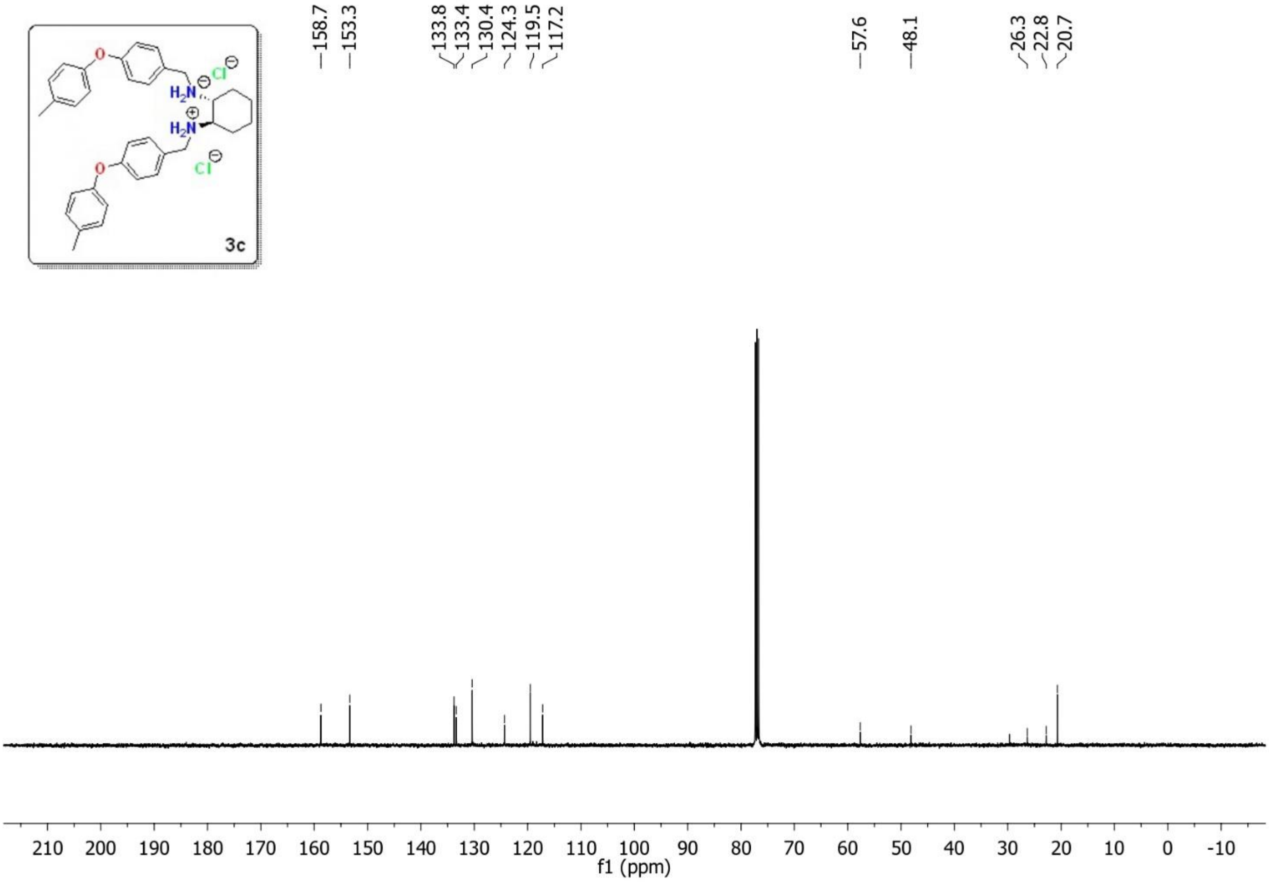

Figure S5. ^{1}H and ^{13}C NMR spectra of compound **3c**

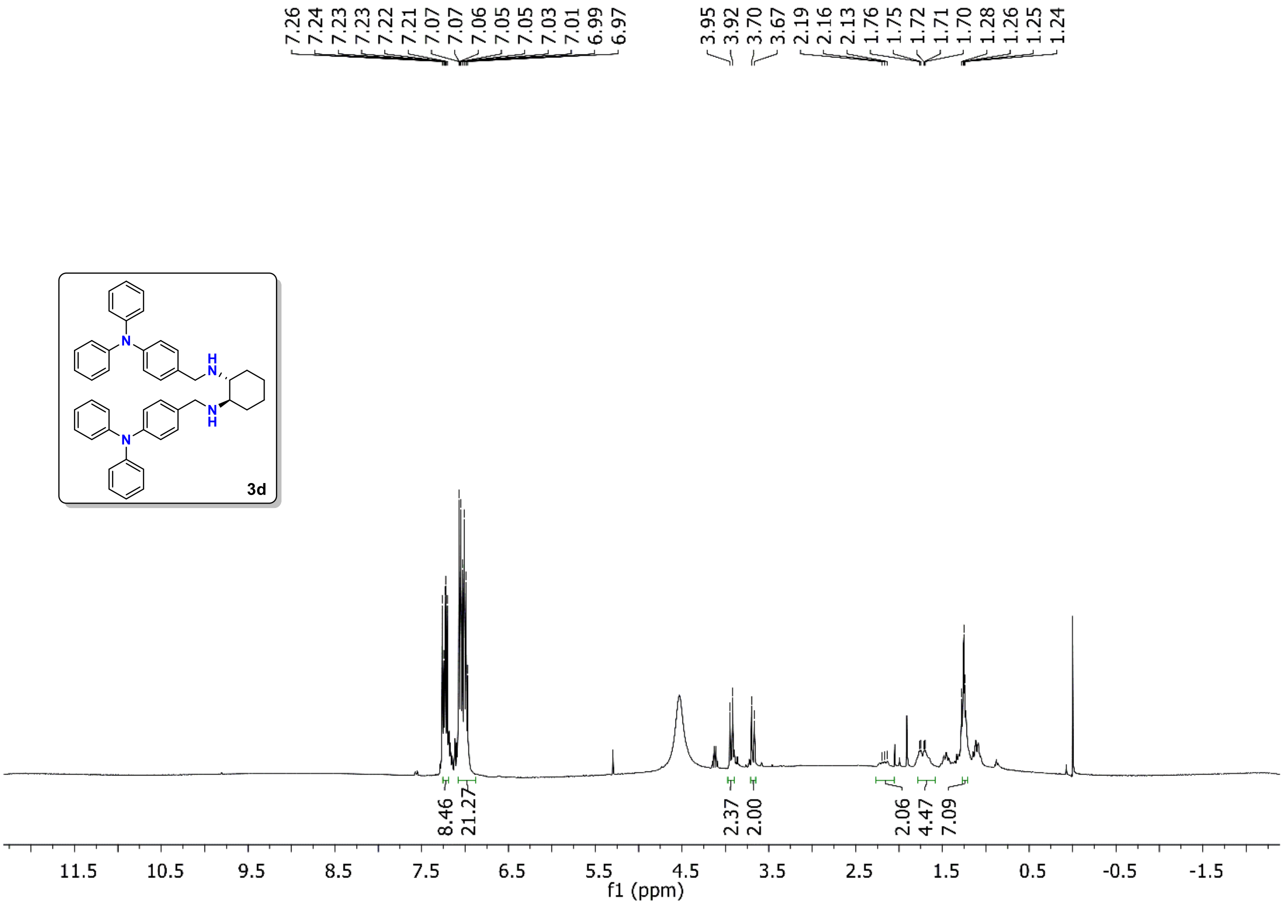

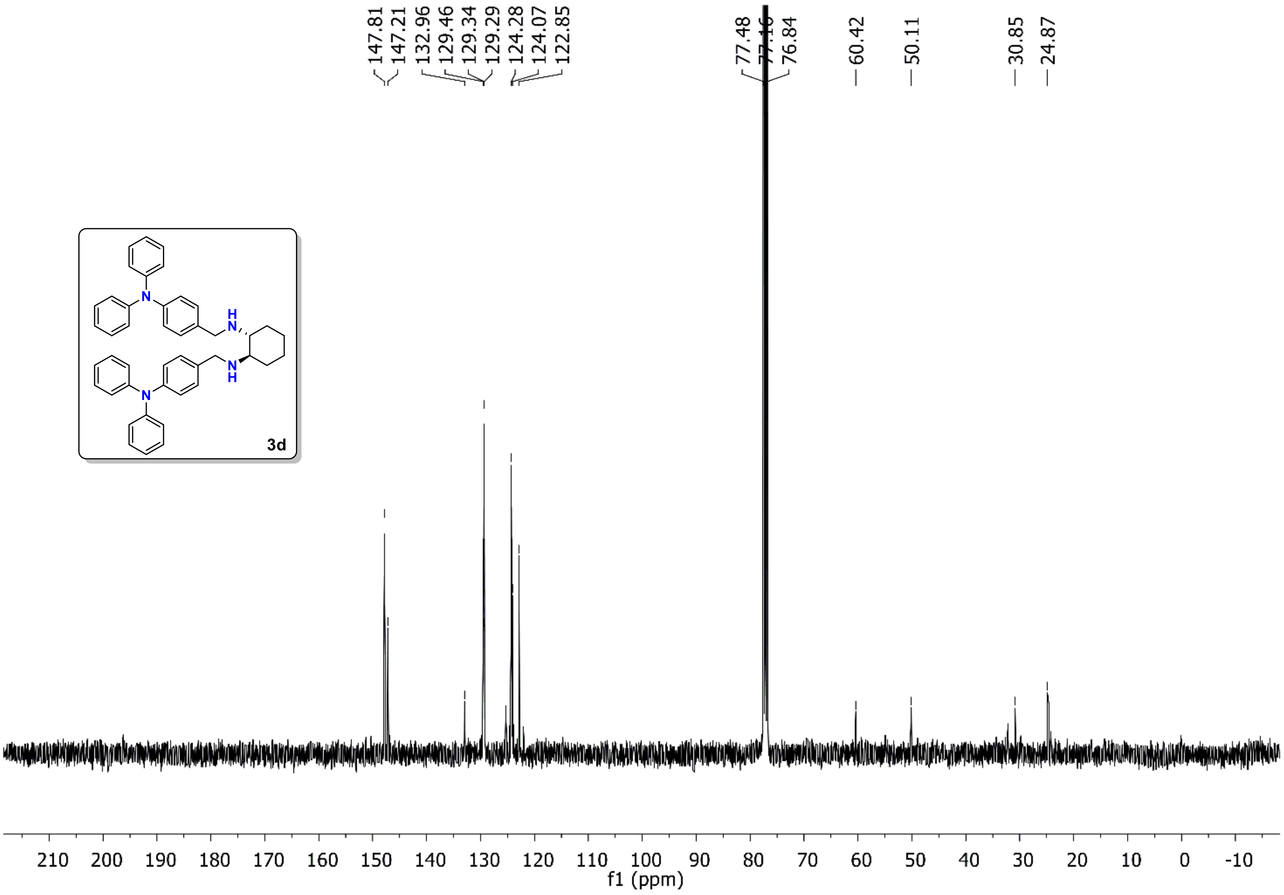

Figure S6. ^{1}H and ^{13}C NMR spectra of compound **3d**

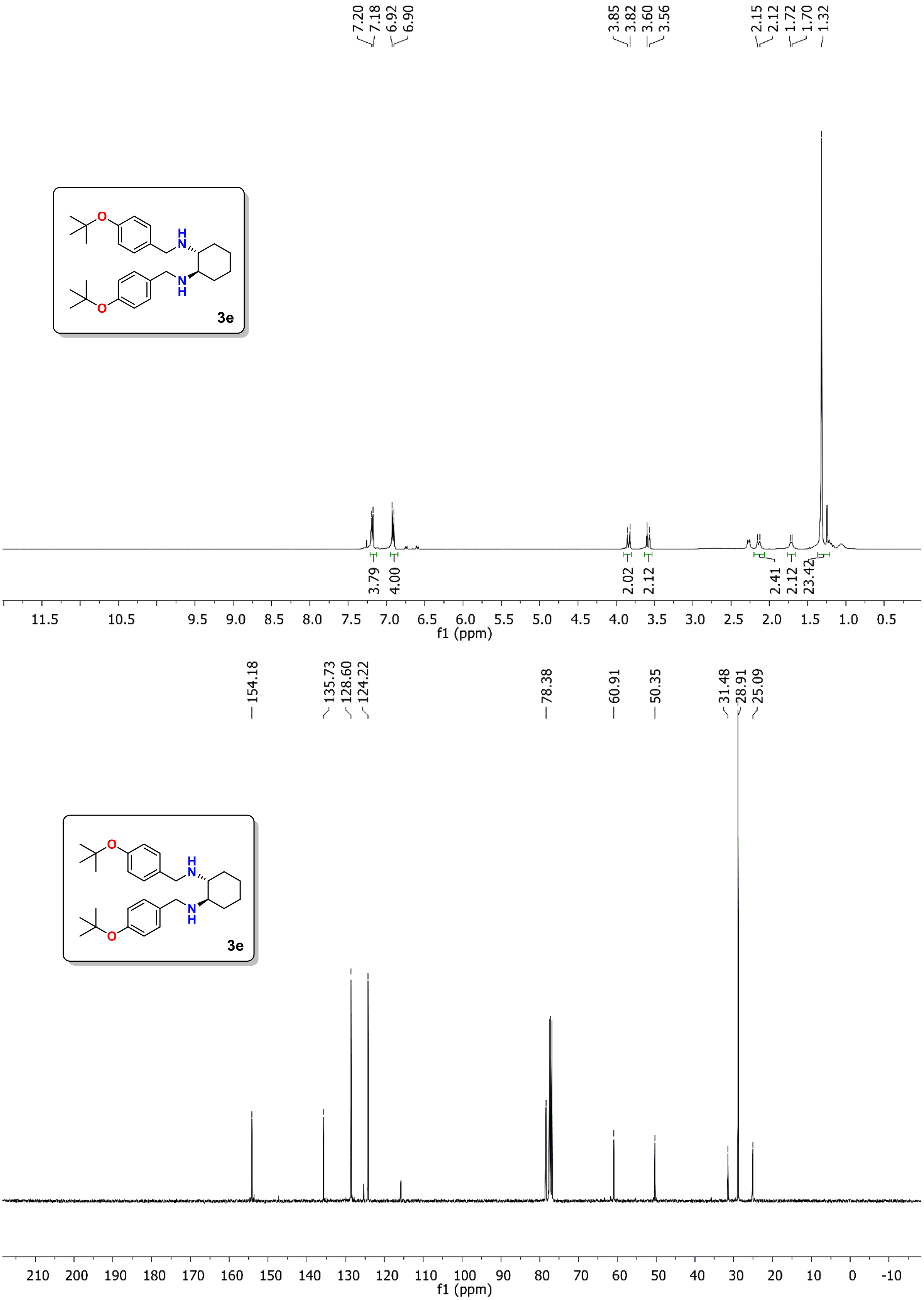

Figure S7. ^{1}H and ^{13}C NMR spectra of compound **3e**

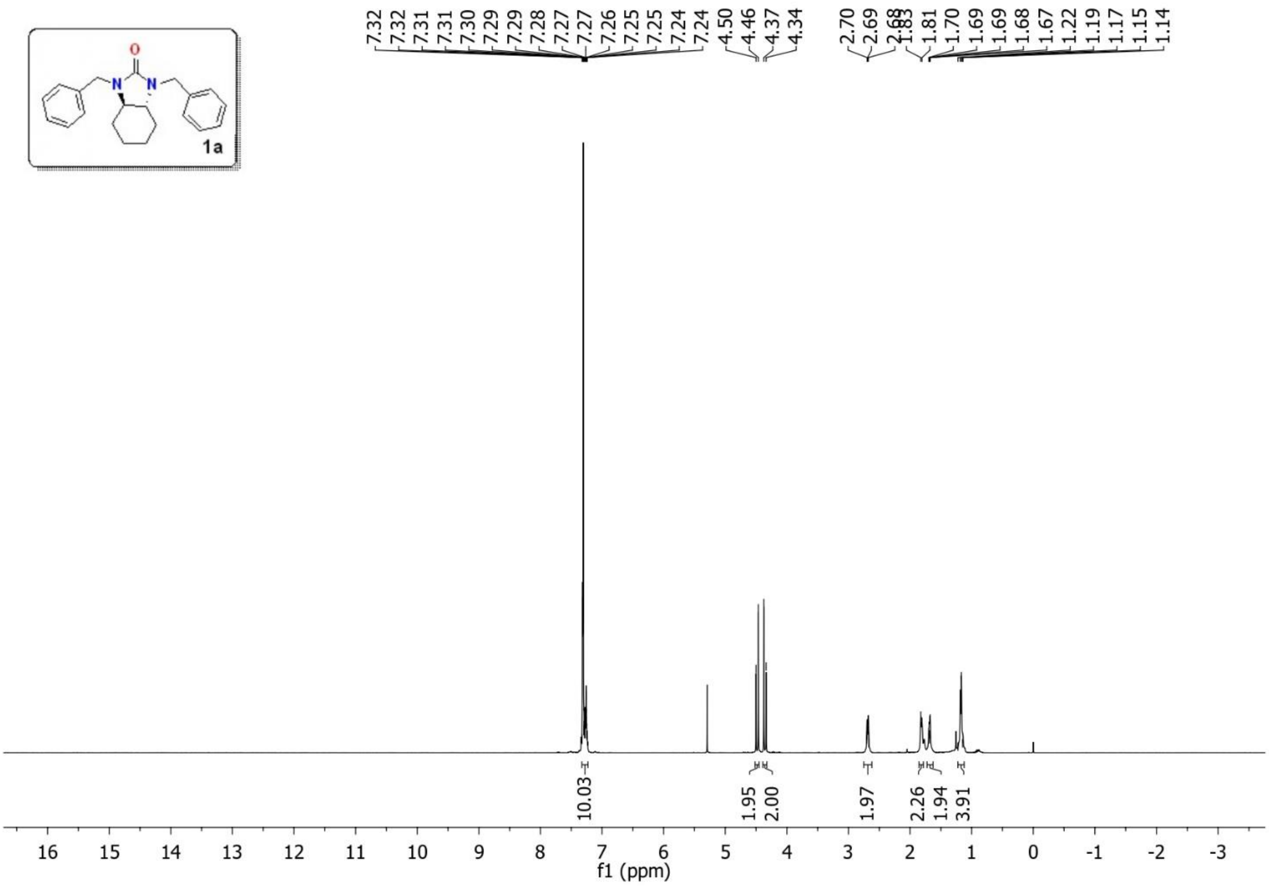

Figure S8. ^{1}H and ^{13}C NMR spectra of compound **1a**

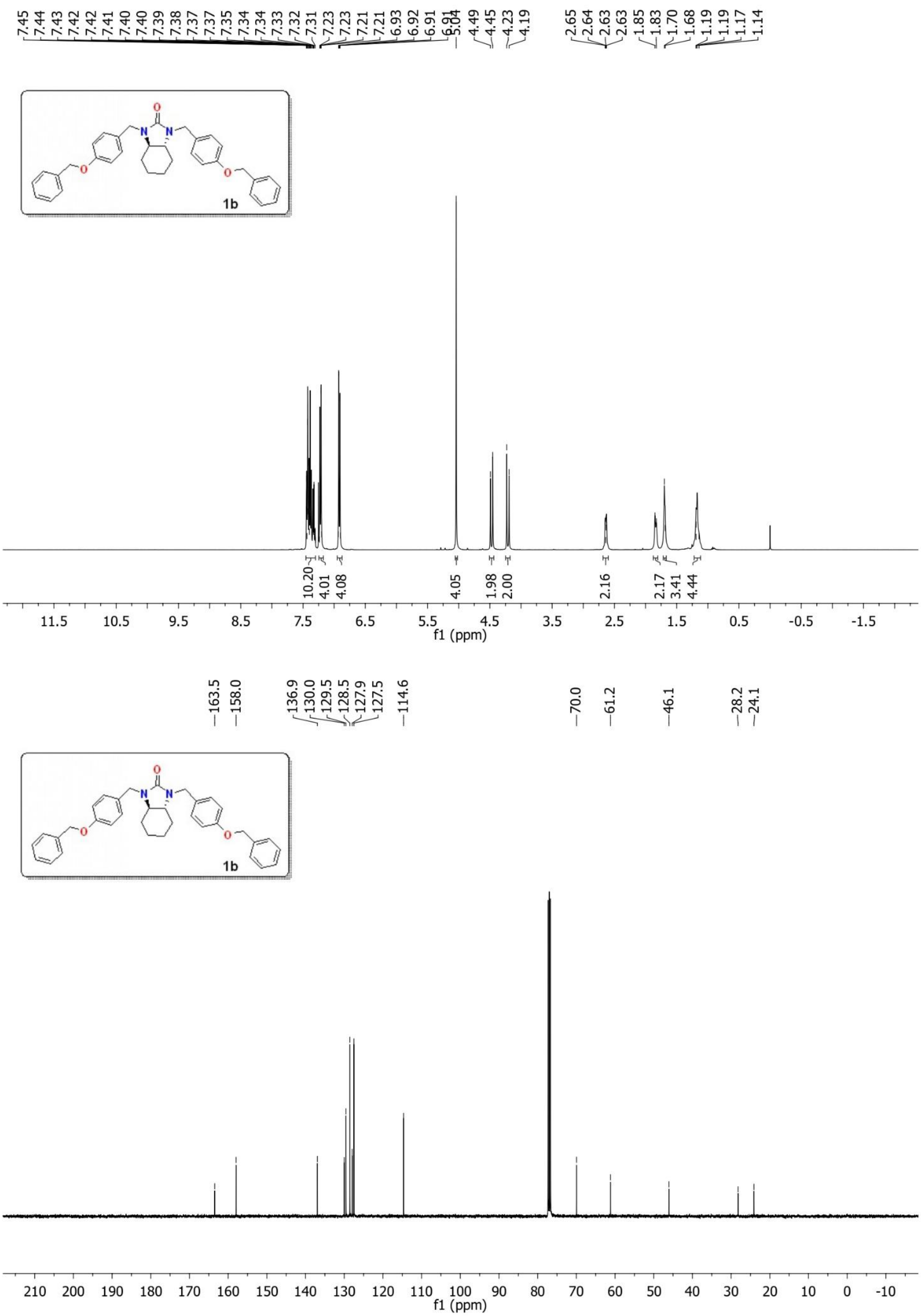

Figure S9. ^{1}H and ^{13}C NMR spectra of compound **1b**

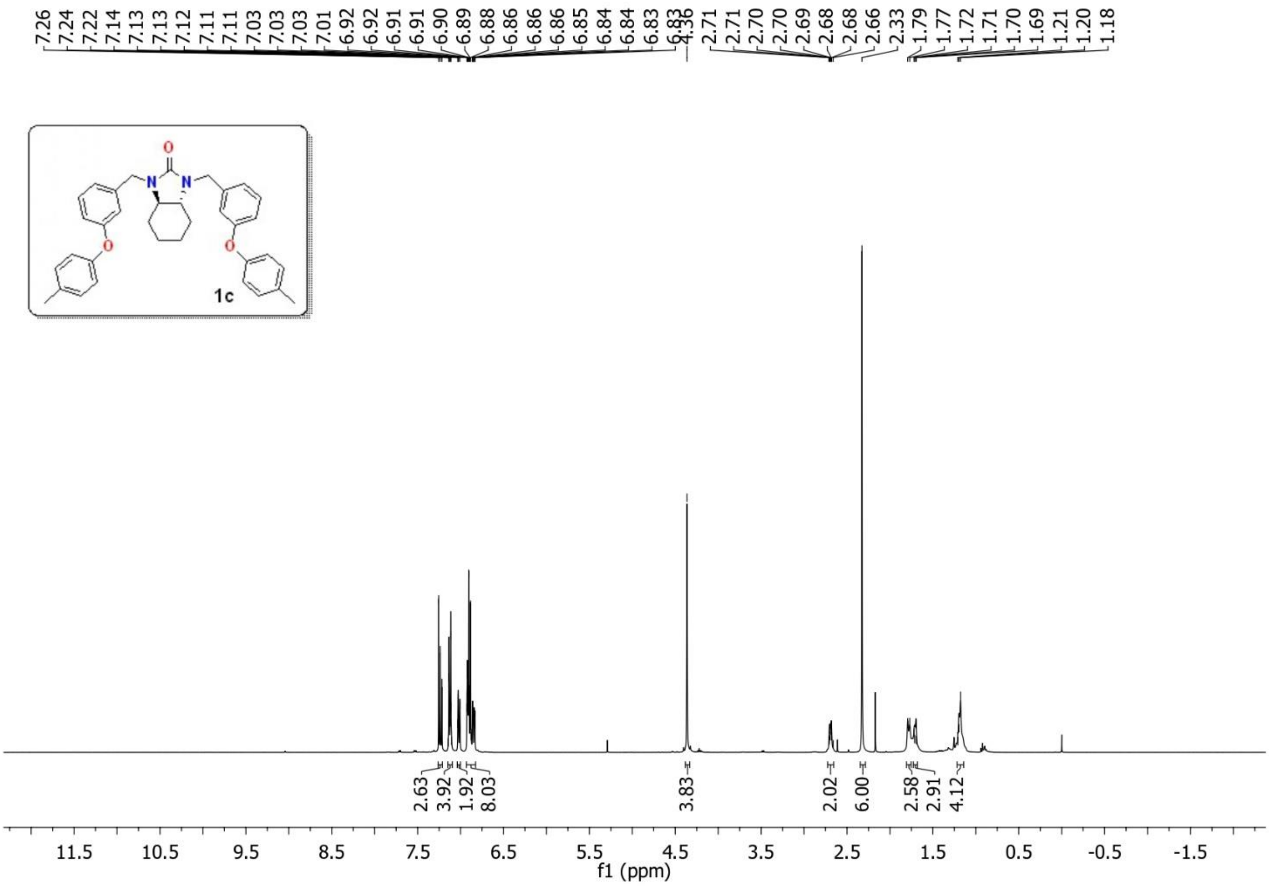

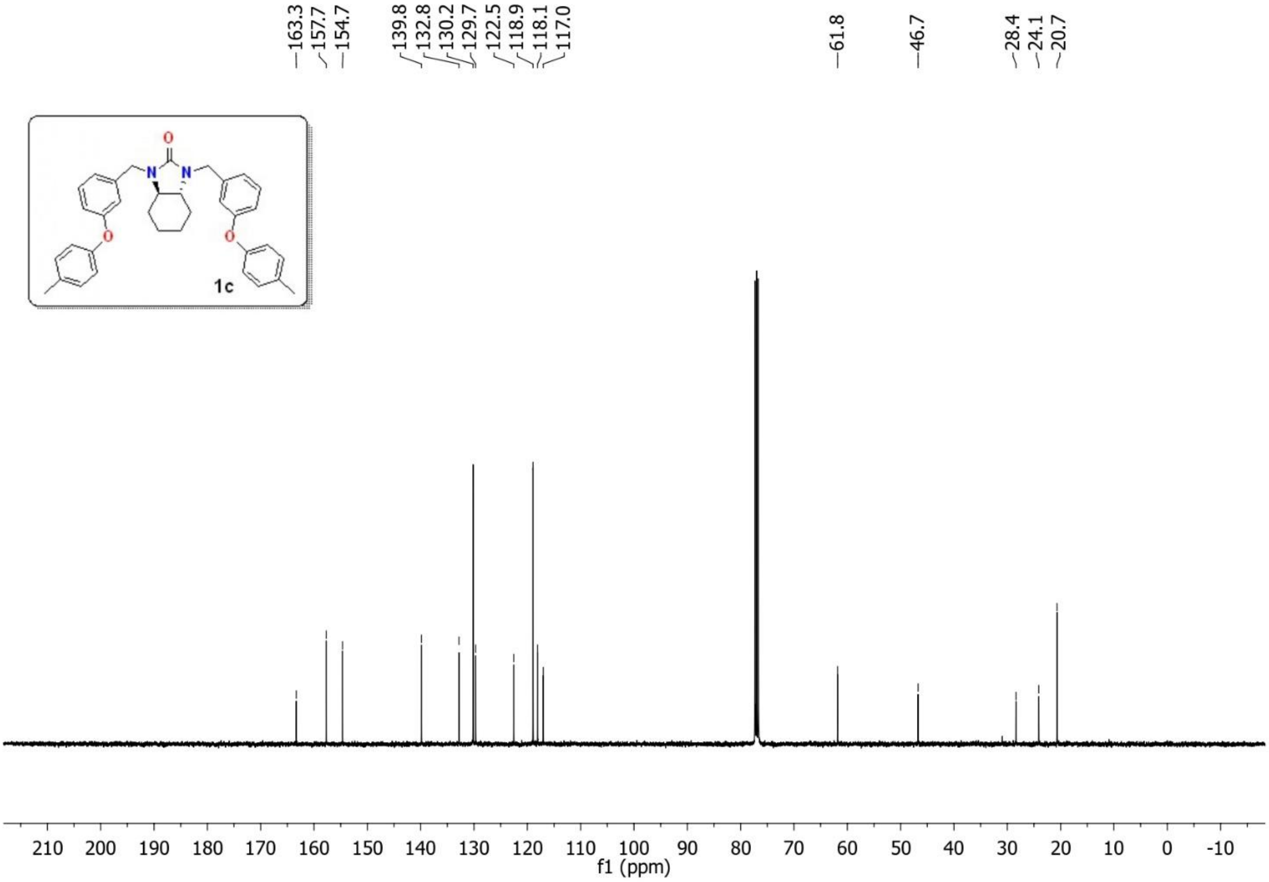

Figure S10. ^{1}H and ^{13}C NMR spectra of compound **1c**

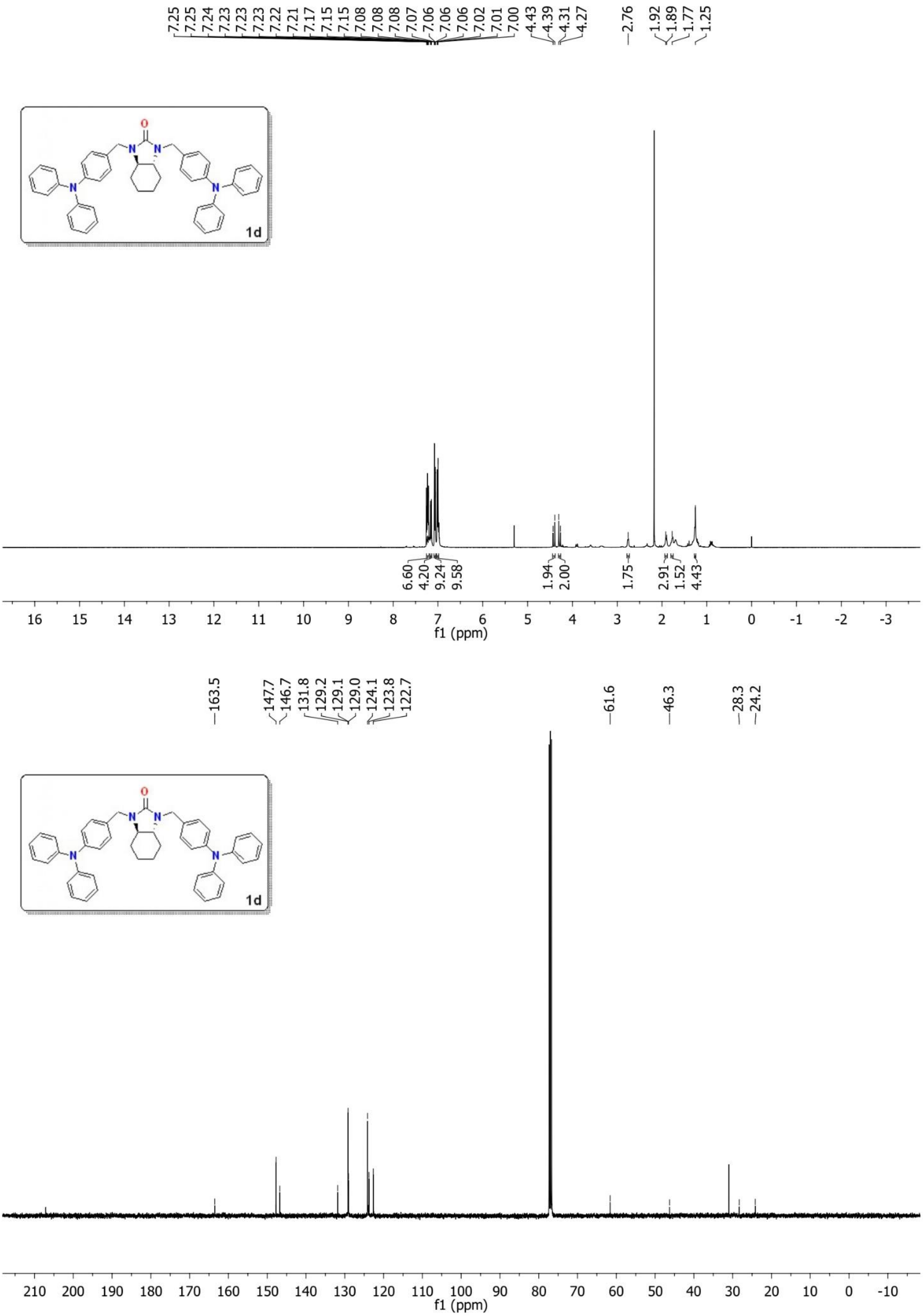

Figure S11. ^{1}H and ^{13}C NMR spectra of compound **1d**

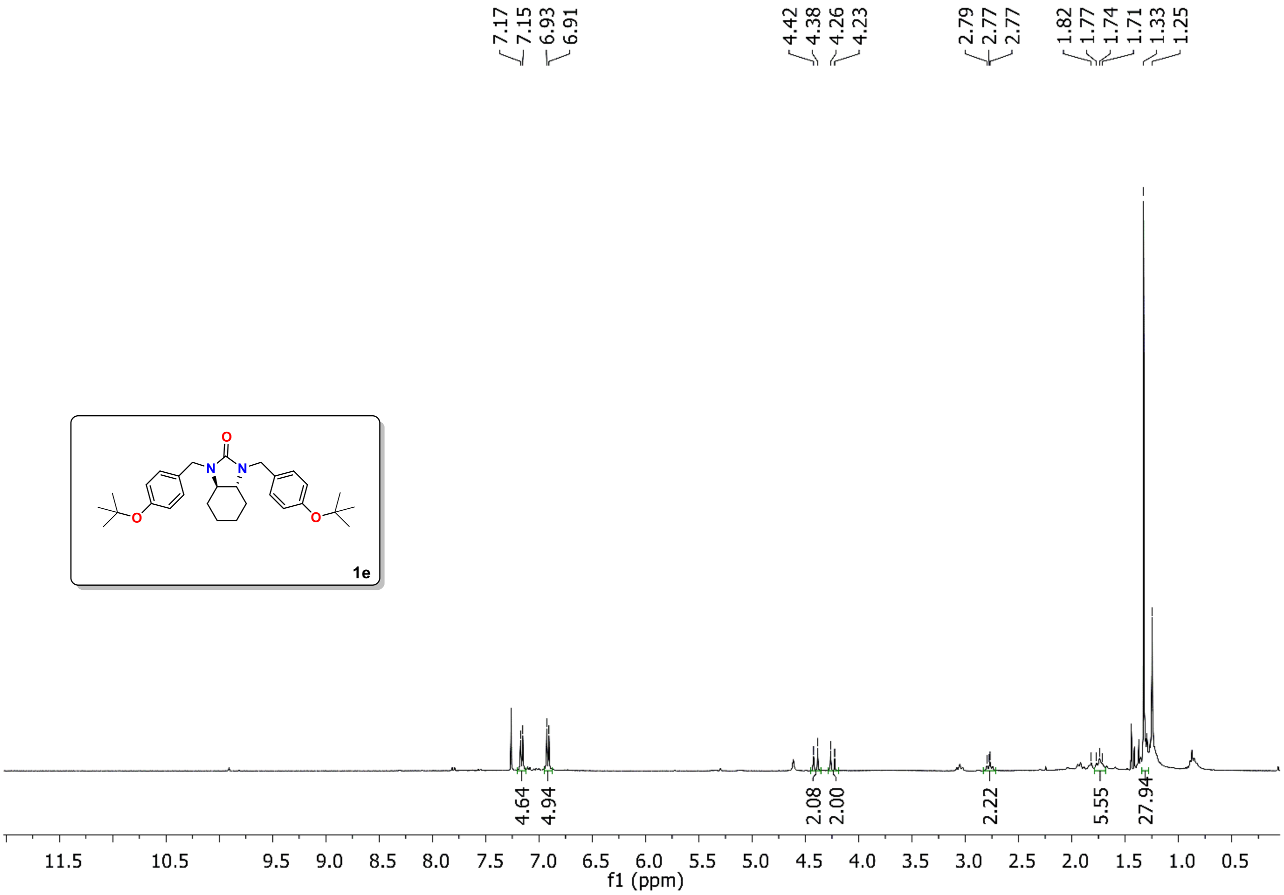

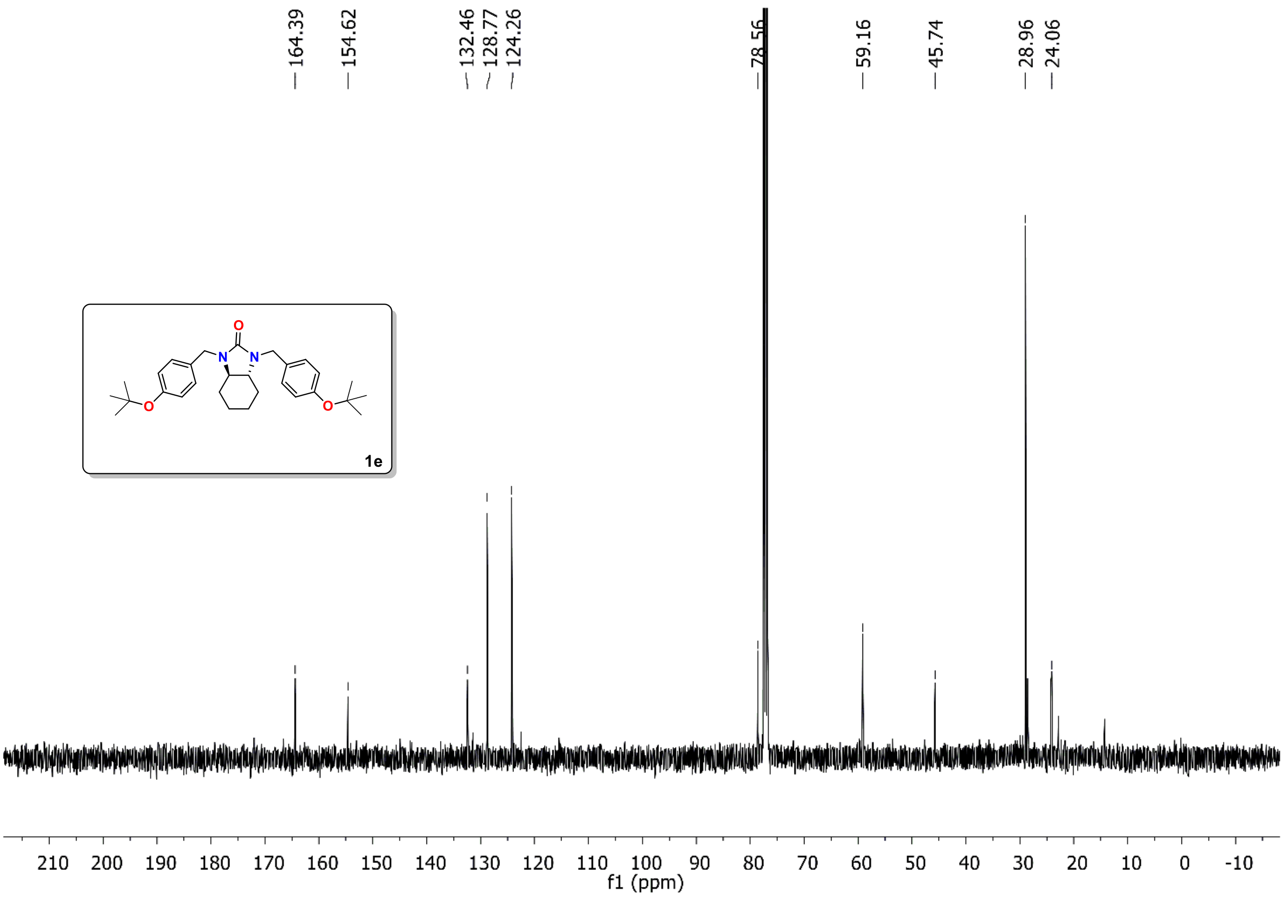

Figure S12. ^{1}H and ^{13}C NMR spectra of compound **1e**

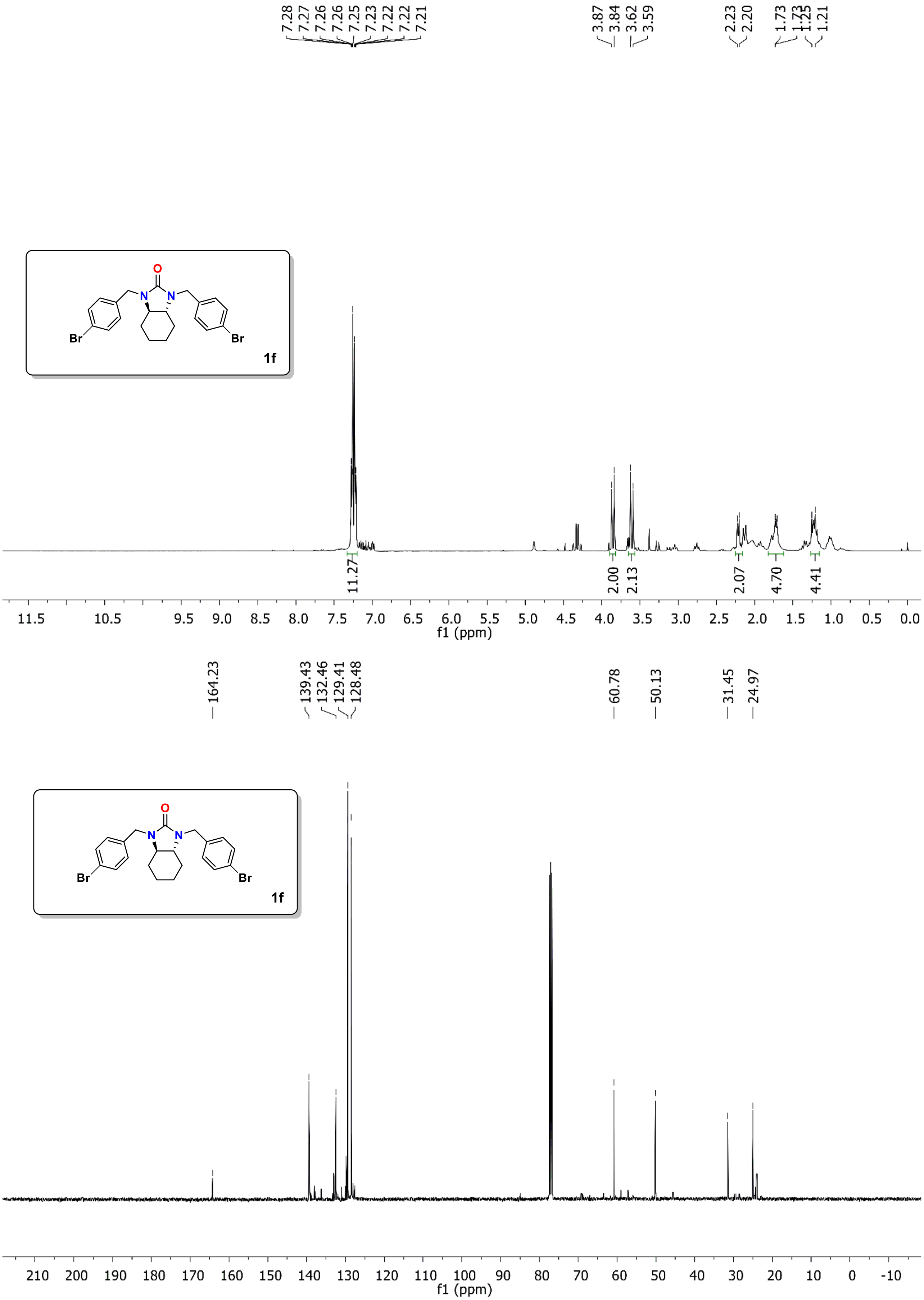

Figure S13. ^{1}H and ^{13}C NMR spectra of compound **1f**

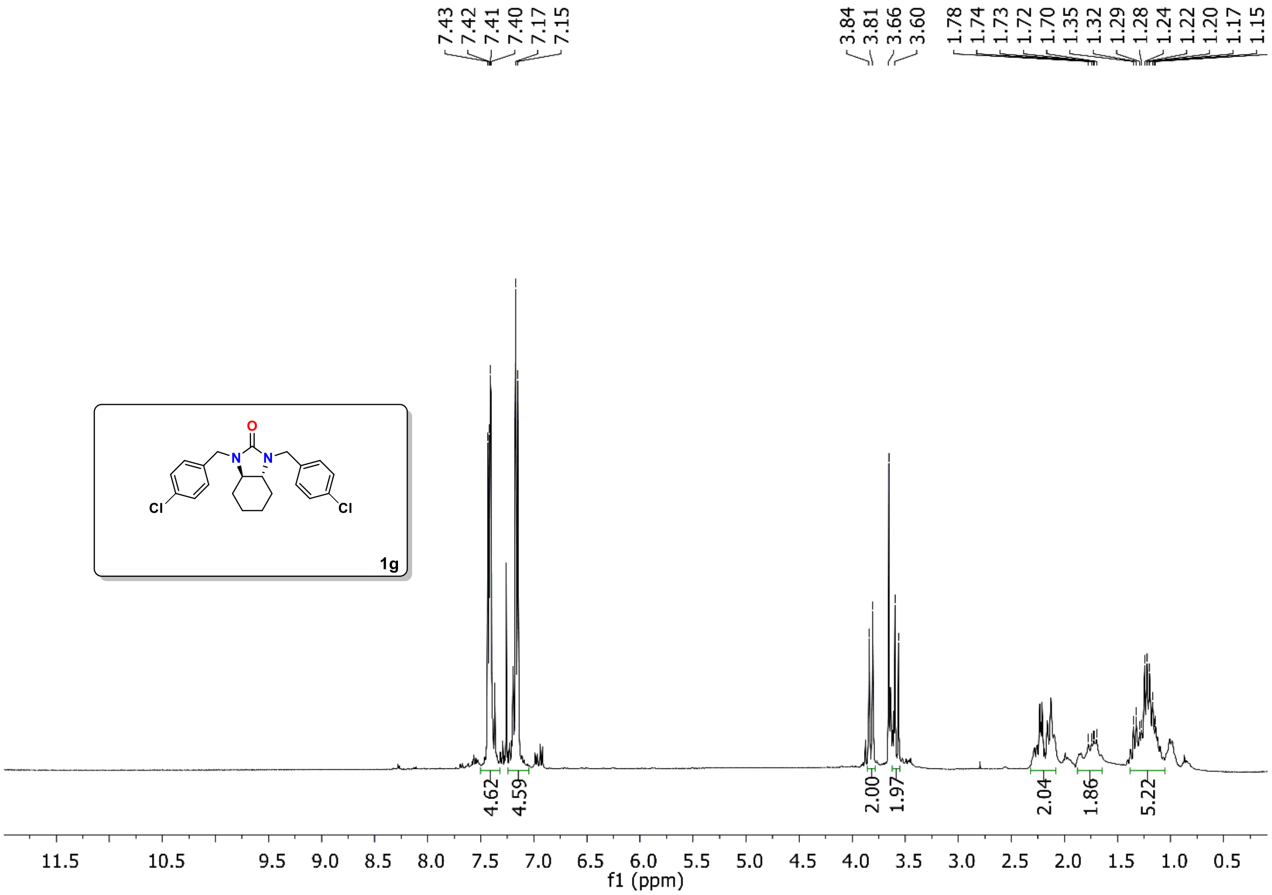

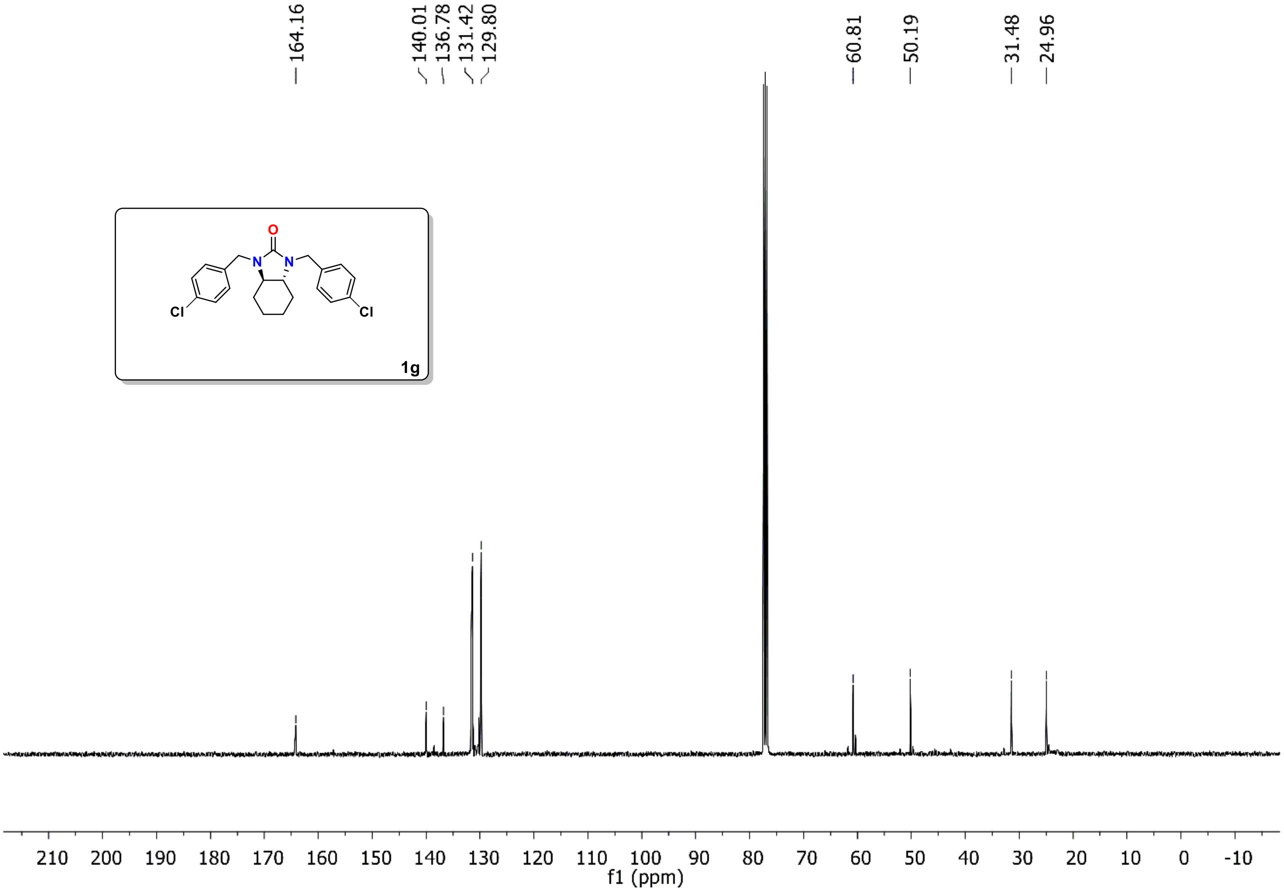

Figure S14. ^{1}H and ^{13}C NMR spectra of compound **1g**

Line#:12 R.Time:----(Scan#:----)
MassPeaks:1665
Spectrum Mode:Averaged 5.805-5.808(3484-3486) Base Peak:321.1911(766051)
BG Mode:Calc Segment 1 - Event 1

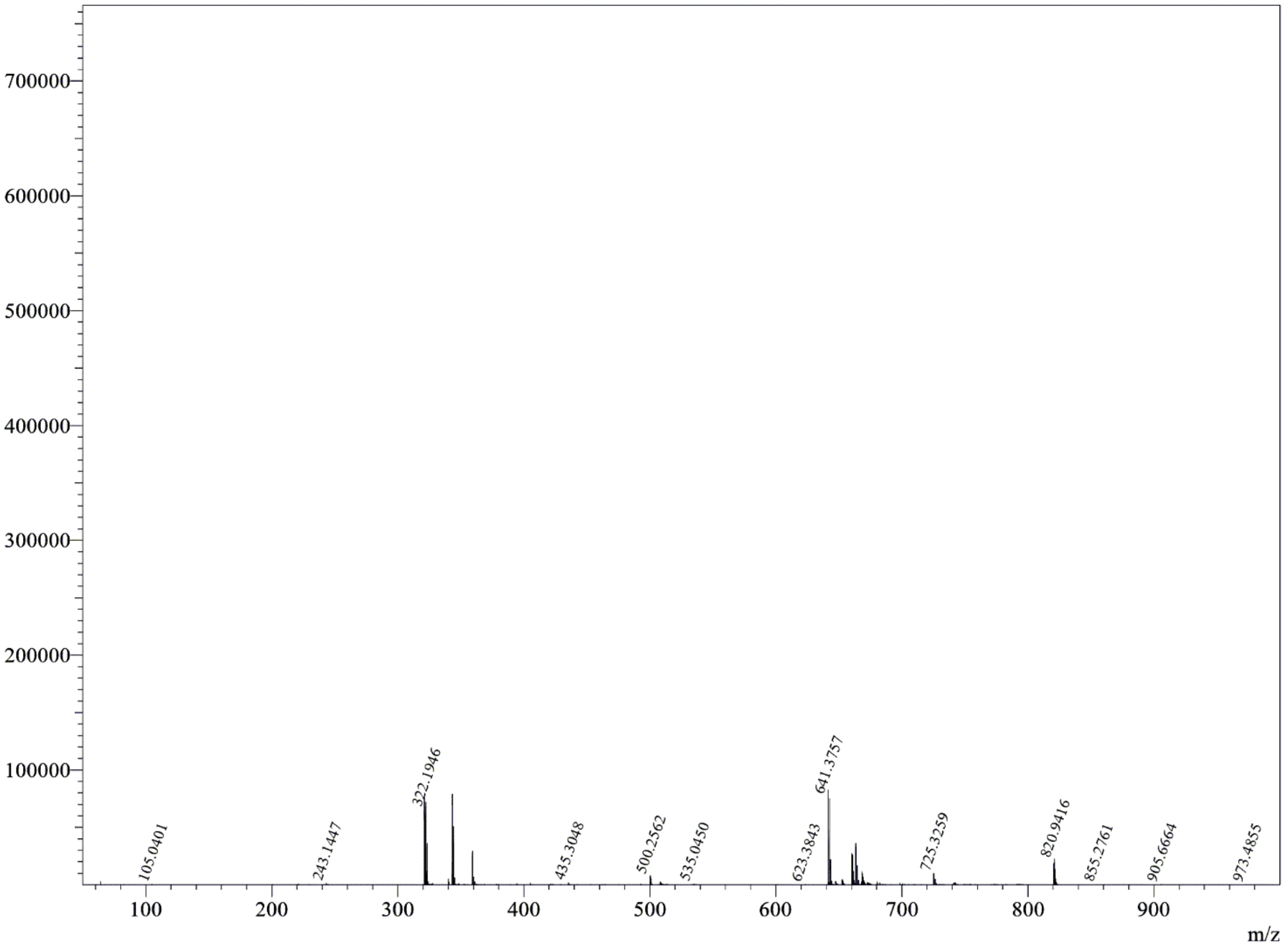

Figure S15. HRMS spectrum of compound **1a**

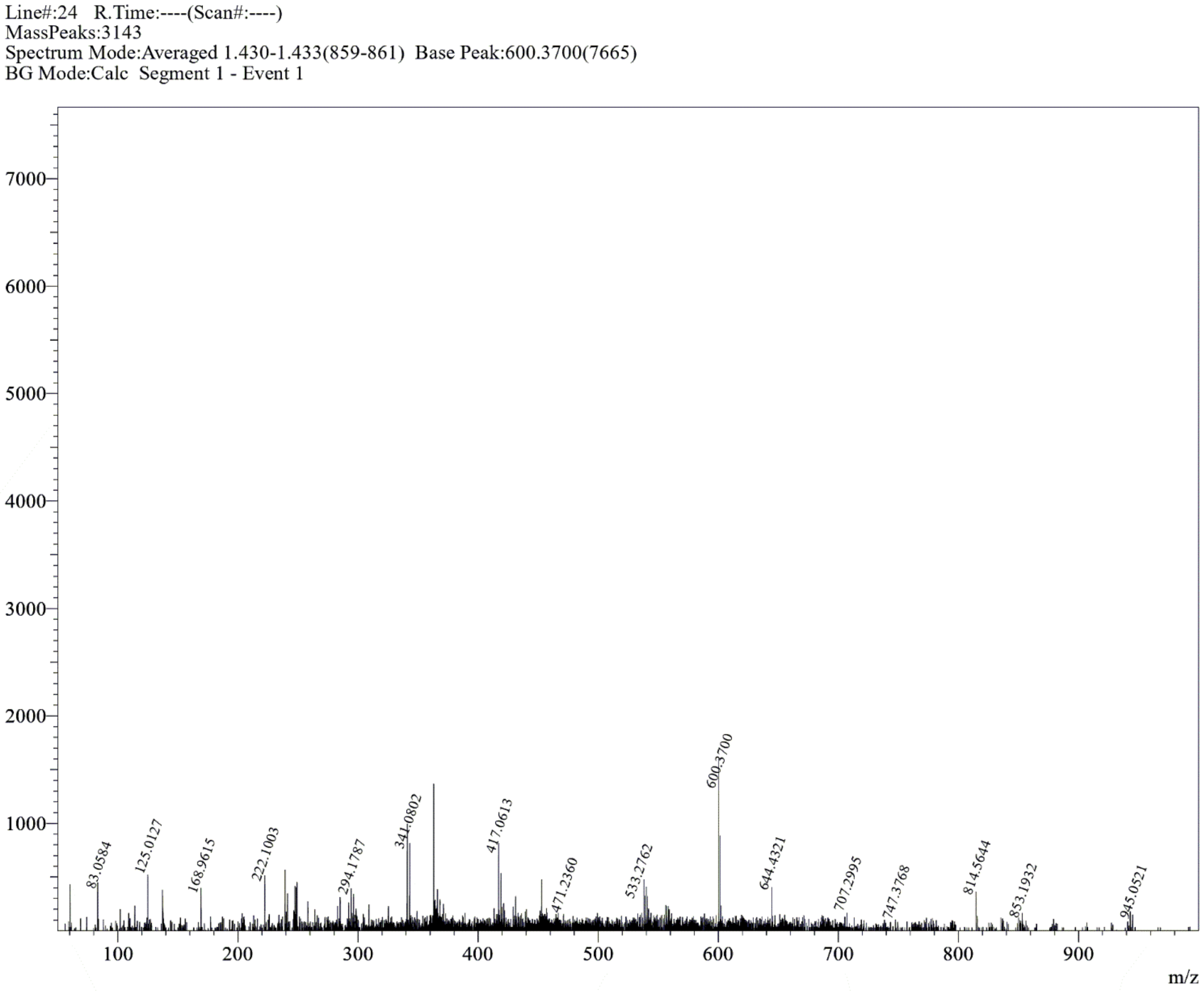

Figure S16. HRMS spectrum of compound **1b**

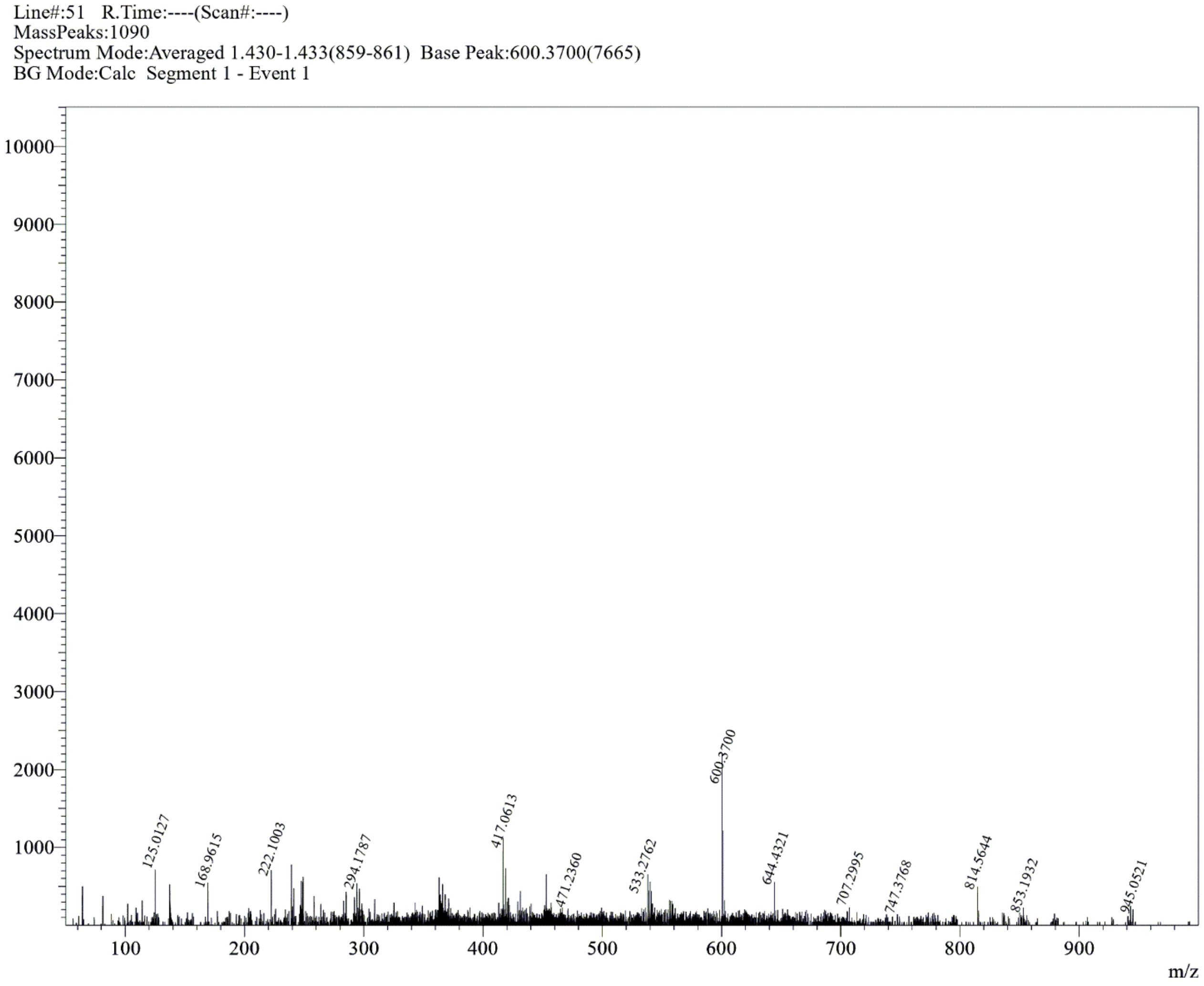

Figure S17. HRMS spectrum of compound **1c**

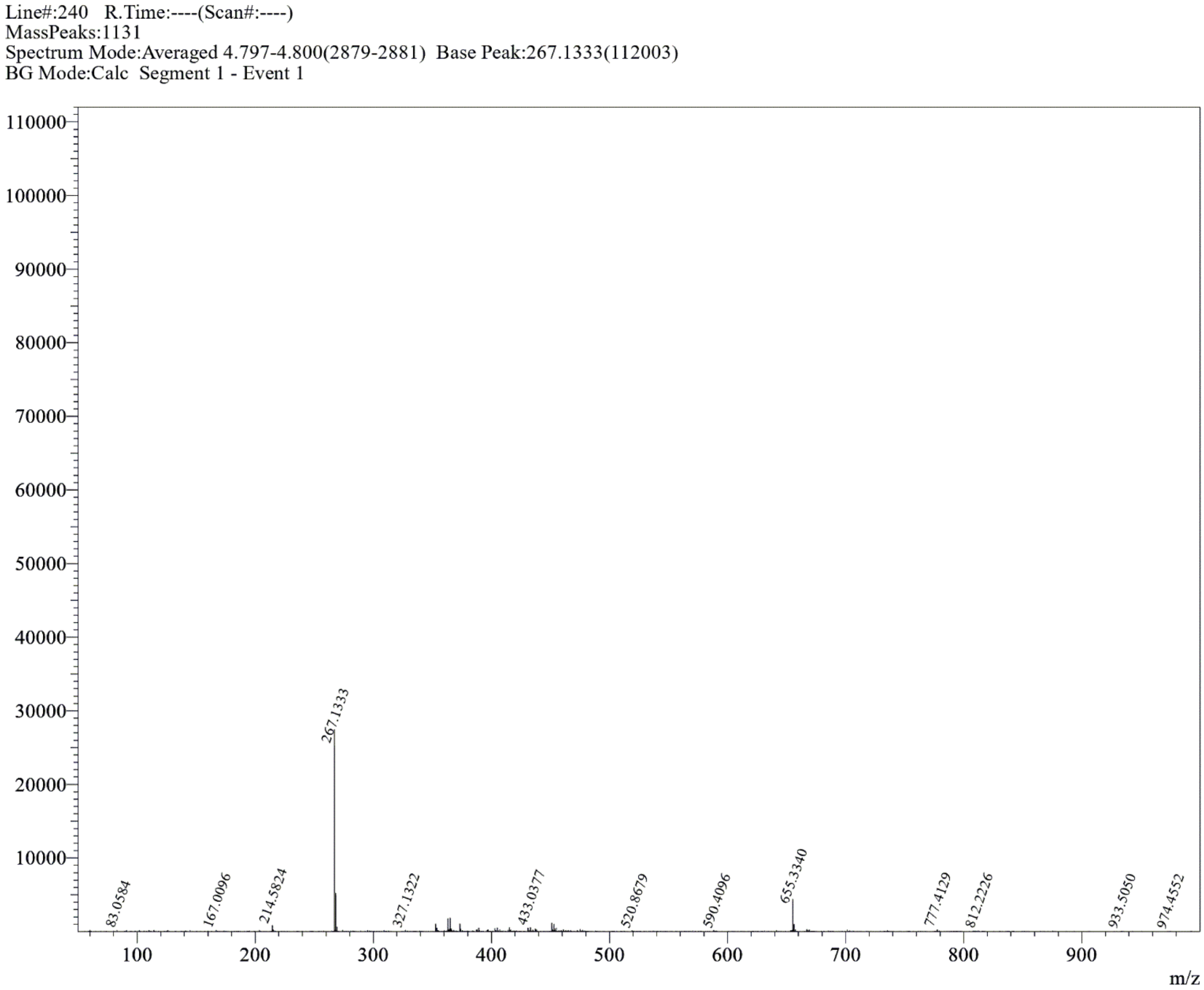

Figure S18. HRMS spectrum of compound **1d**

Line#:126 R.Time:----(Scan#:----)
MassPeaks:655
Spectrum Mode:Averaged 9.870-9.873(5923-5925) Base Peak:321.1909(3284)
BG Mode:Calc Segment 1 - Event 1

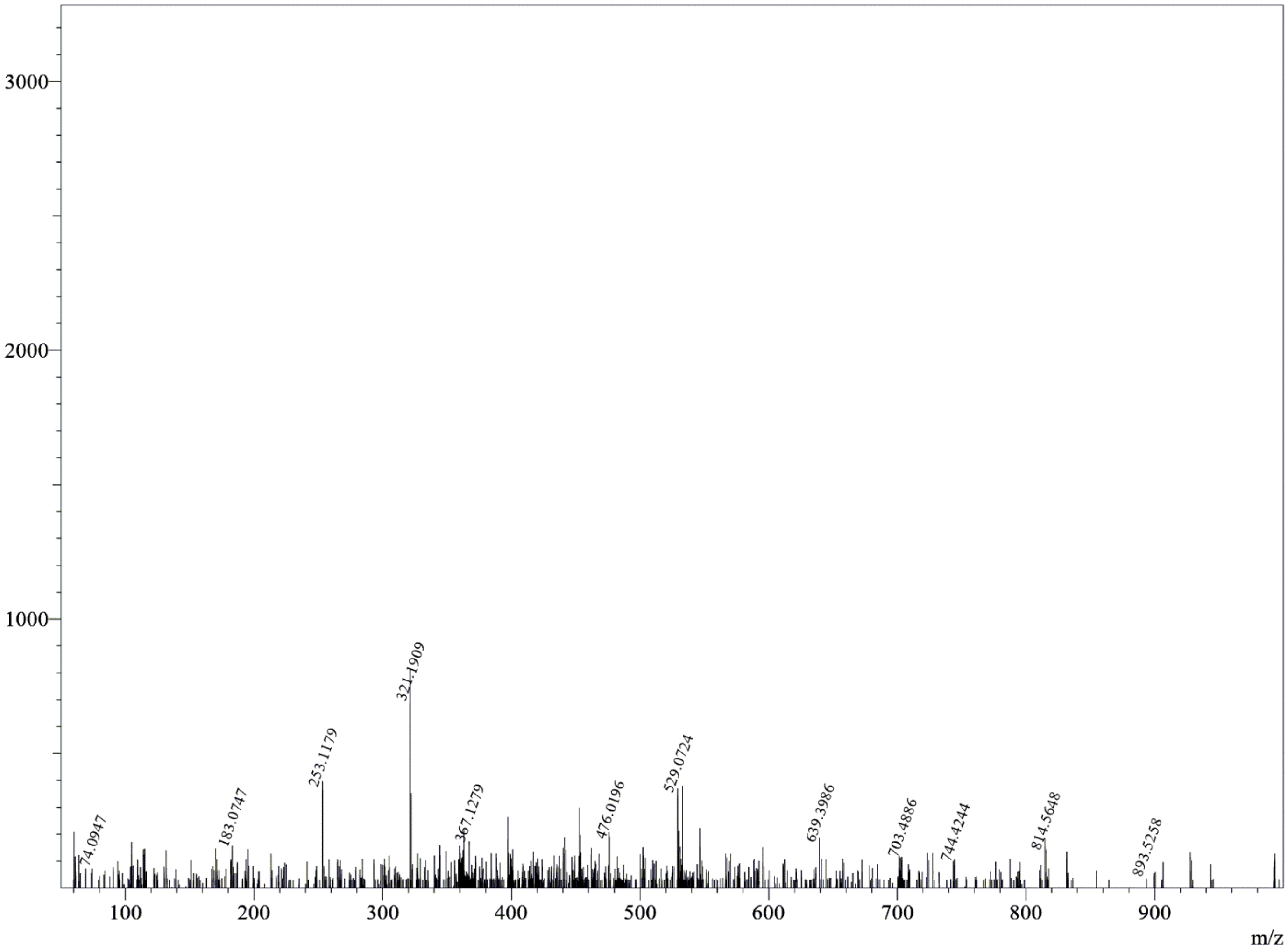

Figure S19. HRMS spectrum of compound **1f**

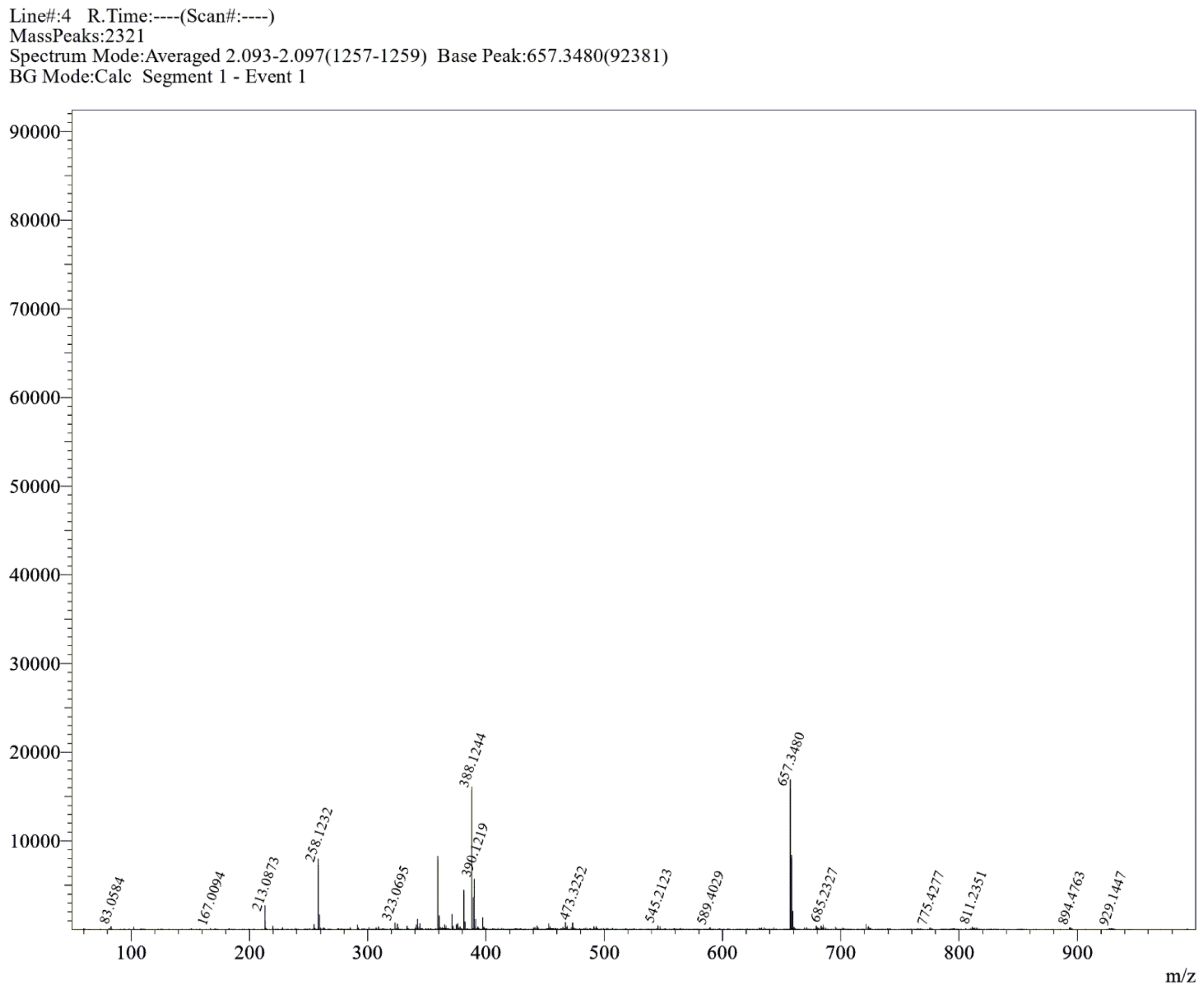

Figure S20. HRMS spectrum of compound **1g**

Table S1. Levels of the factors in the experimental design.

Variable	Units	Level in Coded and Real Values		
		-1	0	+1
Temperature	°C	40	70	100
Time	min	20	40	60
Stoichiometry	/	1	2	3

Table S2. Analysis of Variance (ANOVA) for the Quadratic Model Evaluating the Effects of Independent Variables on Reaction Yield**.**

Source	*Sum of Squares*	*df*	*Mean Square*	*F-value*	*p-value*
**Model**	1.93	9	0.2139	5.53	0.0012
A-Temperature	1.23	1	1.23	31.90	< 0.0001
B-Time	0.1627	1	0.1627	4.21	0.0560
C-Stoichiometry	0.0272	1	0.0272	0.7027	0.4135
AB	0.0217	1	0.0217	0.5602	0.4644
AC	0.0130	1	0.0130	0.3373	0.5690
BC	0.1508	1	0.1508	3.90	0.0648
A^2	0.2335	1	0.2335	6.04	0.0250
B^2	0.0130	1	0.0130	0.3373	0.5690
C^2	0.0698	1	0.0698	1.81	0.1967
**Residual**	0.6574	17	0.0387		
**Cor Total**	2.58	26			

Table S3. Detailed Experimental Design with Observed Response and Tukey Test Results.

Treatment	*Temperature (°C)*	*Time (min)*	*Stoichiometry CDI (eq)*	*Yield (%)*
**1**	40	20	1	18.37 ± 0.13^{fgh}
**2**	40	40	1	34.44 ± 0.10^{d}
**3**	40	60	1	59.53 ± 4.30^{a}
**4**	70	20	1	54.09 ± 1.51^{b}
**5**	70	40	1	61.87 ± 3.52^{a}
**6**	70	60	1	37.17 ± 5.20^{cd}
**7**	100	20	1	14.18 ± 0.06^{hijk}
**8**	100	40	1	7.36 ± 0.90^{mn}
**9**	100	60	1	7.95 ± 0.54^{lmn}
**10**	40	20	2	28.53 ± 0.04^{e}
**11**	40	40	2	59.66 ± 0.03^{a}
**12**	40	60	2	32.46 ± 0.05^{de}
**13**	70	20	2	12.79 ± 0.67^{ijkl}
**14**	70	40	2	18.93 ± 1.29^{fgh}
**15**	70	60	2	15.63 ± 0.23^{ghi}
**16**	100	20	2	8.98 ± 0.44^{klmn}
**17**	100	40	2	10.31 ± 0.34^{jklm}
**18**	100	60	2	9.61 ± 0.44^{jklmn}
**19**	40	20	3	19.17 ± 0.42^{fgh}
**20**	40	40	3	28.33 ± 2.96^{e}
**21**	40	60	3	42.40 ± 0.50^{c}
**22**	70	20	3	20.51 ± 0.12^{fg}
**23**	70	40	3	21.35 ± 0.31^{f}
**24**	70	60	3	61.13 ± 0.25^{a}
**25**	100	20	3	4.60 ± 0.37^{n}
**26**	100	40	3	14.55 ± 0.34^{hij}
**27**	100	60	3	19.28 ± 0.06^{fgh}

Table S4. Specific rotation of precursor **3a-g** and products **1a-g**

**Precursor**	$[\alpha]_D^{25}$	**Product**	$[\alpha]_D^{25}$
**3a**	-38.01°± (c 0.50, MeOH)	**1a**	+8.21°± 0.01 (c 0.01 DCM)
**3b**	-23.71°± 0.01 (c 0.01, DCM).	**1b**	+0.75°± 0.01 (c 0.01 DCM)
**3c**	-23.93°± 0.01 (c 0.01, DCM)	**1c**	+5.49°± 0.01 (c 0.01 DCM)
**3d**	-13.14°± 0.01 (c 0.01, DCM)	**1d**	+13.14°± 0.01 (c 0.01 DCM)
**3e**	-35.81°± 0.01 (c 0.01, DCM)	**1e**	+60.45°± 0.01 (c 0.01 DCM).
**3f**	-12.34° ± 0.02 (c 0.01, MeOH)	**1f**	+44.25°± 0.02 (c 0.01 DCM).
**3g**	-14.11° ± 0.02 (c 0.01, MeOH)	**1g**	+31.17°± 0.02 (c 0.01 DCM).
